# Preparation of Biocomposite Adsorbent Spheres from Bioglass Reinforced Biopolymer Alginate and Adsorption of Crystal Violet

**DOI:** 10.3390/polym18172155

**Published:** 2026-09-03

**Authors:** Aynur Manzak, Yusuf Zandolu, Guler Hasirci, Nilufer Durmaz Hilmioglu

**Affiliations:** 1 Faculty of Science, Department of Chemistry, Sakarya University, Sakarya 54050, Türkiye; yusufzandolu@gmail.com; 2 Faculty of Engineering, Department of Chemical Engineering, Gebze Technical University, Gebze 41400, Türkiye; hasirciguler@gmail.com; 3 Faculty of Engineering, Department of Chemical Engineering, Kocaeli University, Kocaeli 41001, Türkiye; niluferh@kocaeli.edu.tr

**Keywords:** crystal violet, bioglass, alginate, biopolymer

## Abstract

Crystal violet is a cationic dye widely used in biomedical research, industrial dyeing, microbiology, and the textile industry. To remove this toxic dye, which has harmful environmental effects, environmentally friendly composite bioadsorbent spheres were prepared using alginate, a brown seaweed-derived biopolymer, and bioglass. Crystal violet adsorption was performed using a biocomposite adsorbent prepared by adding 14% bioglass to an alginate matrix. The effects of adsorbent amount, dye concentration, contact time, and pH on the adsorption process were investigated. In this study, a maximum removal efficiency of 65.68% was achieved using 40 mg of adsorbent in 20 mL of a 5 mg/L crystal violet solution after 120 min at a stirring speed of 200 rpm. The Langmuir model estimated a theoretical maximum adsorption capacity (qmax) of 5.57 mg/g based on the three investigated initial concentrations (5, 10, and 15 mg/L). The data were analyzed using Response Surface Methodology. ANOVA indicated that contact time had the greatest effect on removal efficiency. Isotherm and kinetic studies revealed that the Langmuir and Dubinin–Radushkevich isotherm models adequately described the adsorption process, and that the second-order kinetic model provided the best fit. These findings suggest that the adsorption is consistent with monolayer adsorption on a relatively homogeneous surface, and that binding may occur via low-energy physical interactions within micropores. The developed bioadsorbents demonstrated promising performance for crystal violet removal.

## 1. Introduction

The contamination of water resources with dyes and organic compounds originating from industries such as textiles, paper, and leather constitutes a global environmental problem because these pollutants can be toxic and carcinogenic even at very low concen-trations and can cause serious damage to aquatic ecosystems [1,2,3].

Crystal violet, methylene blue, rhodamine B, methyl violet, and malachite green are among the common cationic dyes discharged into water bodies [3,4]. Synthetic dyes prevent sunlight from penetrating water bodies, thereby disrupting photosynthetic activity and aquatic ecosystems and ultimately causing ecological imbalance [5,6]. Crystal violet (CV) is a synthetic cationic dye containing a toxic triphenylmethane group and is widely used in various industries, including food processing, textiles, biomedical applications, industrial dyeing, and personal care products [7]. Exposure to crystal violet can cause serious health problems, including eye irritation, respiratory disorders, kidney damage, visual impairment, and cancer. Because it is resistant to microbial degradation, it can accumulate and persist in the environment. Its vibrant color harms aquatic life by inhibiting photosynthesis, necessitating its removal from aquatic environments [8,9,10,11].

Various methods, including adsorption, flotation, oxidation/ozonation, biodegradation, ion exchange, solvent extraction, electrochemical processes, and membrane technologies such as reverse osmosis and nanofiltration, have been employed for dye removal [12,13,14,15,16]. Among these techniques, adsorption has attracted considerable attention because of its cost-effectiveness, recovery potential, and ease of application. The effectiveness of adsorption depends largely on the properties of the adsorbent. Granular activated carbon is one of the most widely used adsorbents; however, its application is often limited by high costs associated with its production, regeneration, and replacement [17,18]. Numerous studies have investigated lower-cost alternatives such as sugarcane pulp extract, plum kernels, perlite, natural clay, chitin, chitosan, fly ash, rice husks, peat, banana extract, and orange peel [19,20,21]. Furthermore, improving water quality through the development of suitable nano-adsorbents has become an important research focus [22]. Since adsorbents derived from synthetic polymers may have adverse environmental impacts, naturally derived polymers are increasingly preferred. Consequently, inexpensive, renewable, and environmentally friendly marine-derived biosorbents have attracted considerable attention [23].

Alginate, extracted from brown seaweed, is a natural, water-soluble, biocompatible, and biodegradable polysaccharide. Sodium alginate is a naturally occurring anionic straight-chain polysaccharide composed of repeating units of L-guluronic acid and D-mannuronic acid residues linked by 1–4 glycosidic bonds [24]. It is well known for its pH sensitivity, non-toxicity, biodegradability, safety, and excellent biocompatibility, making it a valuable biopolymer for biomedical, food, pharmaceutical, and environmental applications [24,25,26,27,28].

One of its most important advantages is its renewability. Because alginate has relatively low mechanical strength and limited surface area, it is often combined with additives such as activated carbon, graphene oxide, clay, and metal oxides. Due to its ability to form gels in the presence of divalent cations such as Ca^2+^, alginate is also used in adsorbent production. Incorporating bioglass into an alginate matrix represents an innovative approach [29].

Biomaterials are substances with unique properties, including ceramics, glasses, metals, and polymers. Their mechanical performance can be improved when combined with filler materials such as natural polymers and glass materials [30,31].

Using environmentally friendly inorganic additives like bioglass is an effective way to increase the adsorption efficiency of such materials and make the adsorbent more functional [32]. Bioglass adsorbent is not merely an inorganic additive. It can create adsorption sites through its functional groups and facilitate interaction between the pollutant and the adsorbent by increasing the heterogeneity and roughness of the biopolymer structure. The prepared biocomposite adsorbent can be easily recovered from the environment and reused without requiring complex separation processes after [32,33]. Bioactive glass (bioglass, BG) is a silica-based material containing calcium and, depending on the formulation, phosphorus-rich components that exhibit bone-like behavior and are widely used in biomedical implant applications [34]. Consequently, bioglass is regarded as an advanced biomaterial due to its high level of biocompatibility [35]. In addition to biomedical applications, its porous structure and high surface reactivity indicate considerable potential for environmental applications. Bioglass is available in several forms, including particles, pellets, and powders, and is used as an adsorbent. However, these forms often limit the reusability of adsorbents. Forming composite structures with biopolymers can overcome this limitation. Incorporating bioglass into an alginate matrix increases mechanical strength and produces a synergistic effect by forming new active binding sites. Moreover, simplified synthesis conditions make bioglass production more economical. The Stöber method enables bioglass production using minimal raw materials [36,37]. Structurally, bioglass exhibits small particle dimensions and a high specific surface area (70–130 m^2^/g), depending on the synthesis conditions [37]. It also is highly biocompatible and thermostable, and it degrades in the environment without causing pollution. Furthermore, its negatively charged surface is suitable for adsorbing cationic dyes, making it a promising adsorbent for their removal [38]. Therefore, composite adsorbents combining renewable alginate and mechanically robust bioglass can enhance adsorption capacity and dye removal performance.

Although alginate-based composites are frequently employed in environmental applications, bioglass-based composites are mostly used for biomedical applications. Moreover, reports on bioglass-based adsorbents remain limited. To the best of our knowledge, the removal of crystal violet using sodium alginate/bioglass composite beads has not previously been reported. Furthermore, the contribution of bioglass-induced surface heterogeneity to adsorption performance has not been adequately discussed in the literature. The present study aims to enhance removal efficiency by creating a synergistic effect through combining a biopolymer and bioglass in the resulting adsorbent, leveraging the biopolymer’s functional groups and the surface heterogeneity provided by the bioglass. Accordingly, this study presents the development of a low-cost, environmentally friendly sodium alginate/bioglass biocomposite adsorbent prepared from minimal raw materials.

In this study, bioglass synthesized using a minimal-material approach was incorporated into an alginate matrix. The adsorption of crystal violet onto the synthesized low-cost biocomposite adsorbent was investigated. The effects of adsorbent dosage, initial dye concentration, and contact time—key parameters influencing adsorption performance—were evaluated. Furthermore, the adsorption data were analyzed using equilibrium isotherm and kinetic models. Experimental data were statistically optimized using Response Surface Methodology (RSM).

The production of petroleum-derived synthetic polymers is energy intensive and generates a substantial carbon footprint owing to greenhouse gas emissions. In contrast, alginate, a natural polymer derived from seaweed, has a relatively low carbon footprint because seaweed sequesters carbon through photosynthesis. While hazardous organic solvents are commonly used in the preparation of synthetic polymer-based adsorbents, alginate can be processed in water, a green solvent. Unlike synthetic polymers, which may persist in the environment for hundreds of years and contribute to microplastic pollution, the alginate/bioglass biocomposite developed in this study is biodegradable, decomposing into non-toxic components. Conventional adsorbents are generally derived from synthetic polymers or activated carbon, contributing to fossil resource depletion. In contrast, the use of renewable alginate eliminates the need for fossil-based materials. Moreover, since the alginate/bioglass biocomposite can be prepared at room temperature, energy consumption and fossil fuel use are reduced [36]. These characteristics highlight the significance of the developed adsorbent from the perspectives of green chemistry and energy efficiency.

## 2. Materials and Methods

### 2.1. Materials

Tetraethyl orthosilicate (TEOS, 97.5%), sodium alginate, calcium chloride (97.0%), and calcium nitrate tetrahydrate (99.0%) were obtained from Isolab (Eschau, Germany); ammonium hydroxide, (ammonia solution 30%) was obtained from Carlo Erba Reagents (Milan, Italy); ethanol, HCl (37%), and NaOH were supplied by Merck (Darmstadt, Germany); and crystal violet (0.5%, aqueous solution) was obtained from Kimyalab (Istanbul, Türkiye). Crystal violet (CV) is an industrial dye belonging to the triphenylmethane dye group and contains positively charged functional groups [39]. The properties of crystal violet are summarized in Table S1.

### 2.2. Preparation of Biocomposite Adsorbents

A solution containing 4.5 mL of ammonium hydroxide, 15 mL of water, and 10 mL of ethanol was stirred for 10 min. Separately, a mixture of 25 mL of ethanol and 3 mL of TEOS was stirred for 10 min. The two solutions were combined and stirred for 30 min; 1.59 g of calcium nitrate tetrahydrate was then added, and the mixture was stirred for another 90 min. The resulting suspension was centrifuged at 3000 rpm for 10 min. The precipitate was washed twice with water and once with ethanol, scraped into a Petri dish, dried overnight in an oven at 60 °C, and finally calcined at 700 °C for 2 h at a heating rate of 2 °C/min [36].

**Figure 1:**
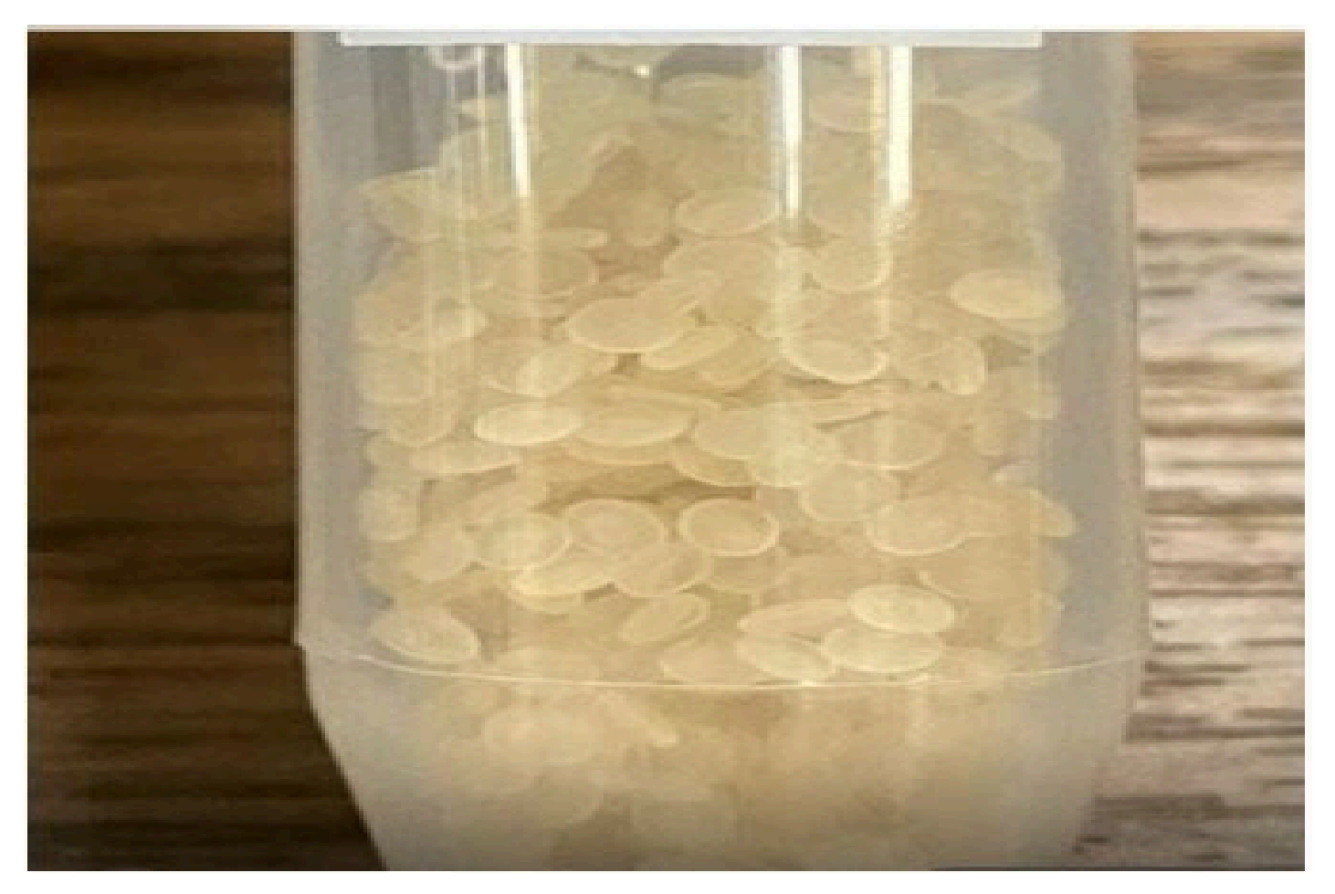
Bioglass-modified adsorbent beads.

Sodium alginate (5 g) was added to 95 mL of hot water and sonicated in an ultrasonic bath operating at 35 kHz for 30 min to obtain a homogeneous biopolymer solution. Bioglass powder, corresponding to 14 wt% of the sodium alginate content, was dispersed in a small volume of water by ultrasonication and gradually added to the alginate solution by the priming technique. The biocomposite mixture was stirred until a homogeneous suspension was obtained, then transferred to a syringe and added dropwise into a coagulation bath containing 500 mL of 50 mmol CaCl_2_ solution. The resulting adsorbent spheres were cured in the coagulation bath with stirring for 24 h. After washing with water for neutralization, the beads were dried at room temperature for 3 days. The prepared bioglass-modified adsorbent beads are shown in Figure 1.

### 2.3. Characterization of Adsorbents

The dye concentration in the adsorption experiments was determined using a UV–Vis spectrophotometer (Shimadzu UV-2600; Shimadzu Corporation, Kyoto, Japan). The solution pH was measured using an Schott CG 840 pH meter (Schott AG, Mainz, Germany). The surface morphology of the biocomposite was examined by scanning electron microscopy (SEM; JEOL JSM-6060LV; JEOL Ltd., Tokyo, Japan). The crystal structure was analyzed using X-ray diffraction (XRD; Rigaku D/MAX/2200/PC; Rigaku Corporation, Tokyo, Japan). The textural characterization of the biocomposite was determined using a surface area and pore size analyzer (Autosorb-1C/MS; Quantachrome Instruments, Boynton Beach, FL, USA) based on N_2_ adsorption at 77 K. Transmission electron microscopy (TEM) was performed using a high-contrast FEI Tecnai CTEM microscope (FEI Company, Hillsboro, OR, USA).

### 2.4. Batch Adsorption Studies

**Figure 2:**
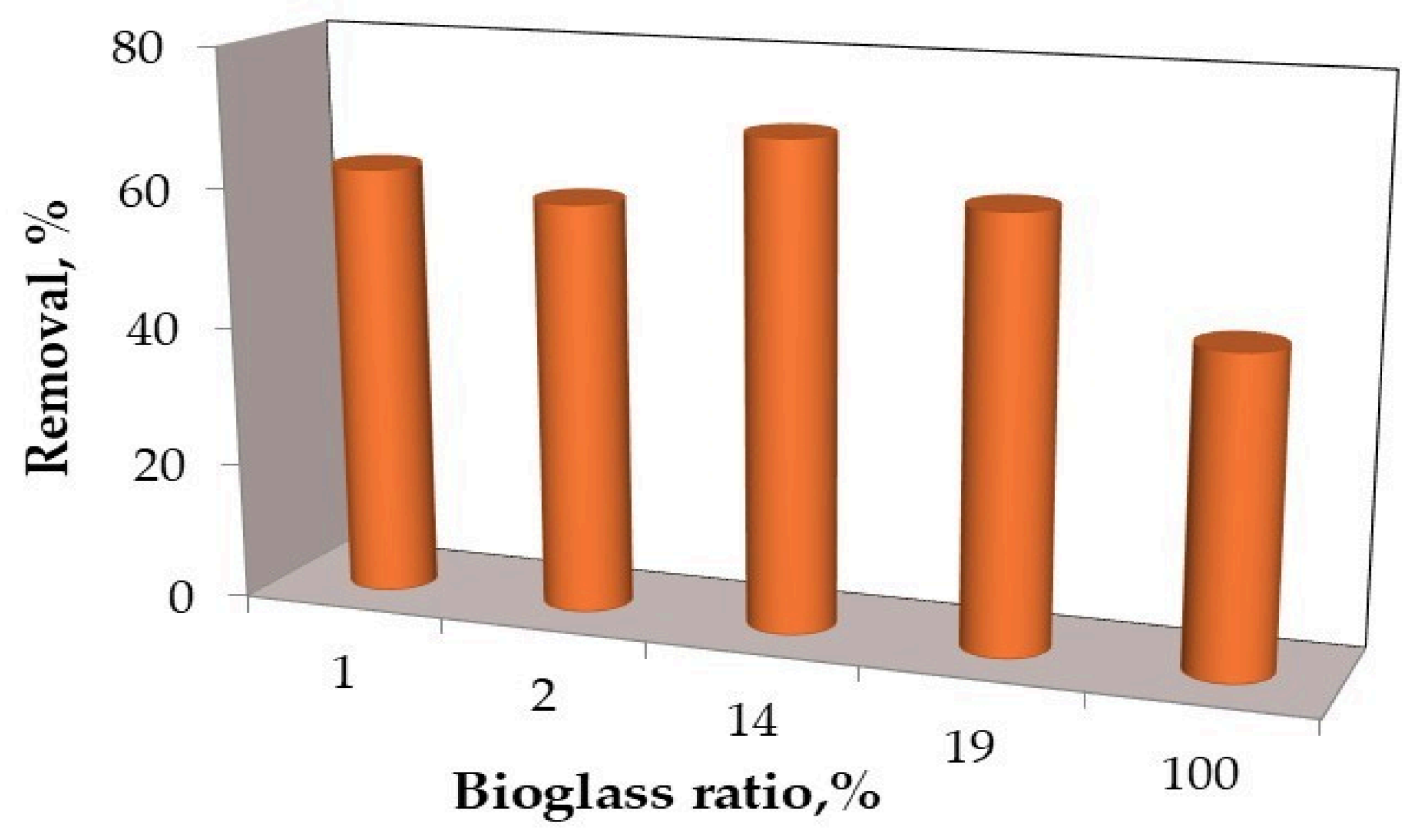
The relationship between dye removal efficiency and bioglass ratio.

Bioglass was incorporated into alginate at concentrations of 1%, 2%, 14%, 19%, and 100%. As shown in Figure 2, the highest removal efficiency was obtained with 14% bioglass. Therefore, all adsorption experiments were conducted using alginate beads containing 14% bioglass. At this concentration, a suitable balance was established between the active groups and the improved morphological structure provided by bioglass and the functionality of the biopolymer [32]. At this concentration, the sodium alginate matrix may have prevented the aggregation of bioglass nanoparticles [40]. At higher bioglass concentrations, particle agglomeration and reduced accessibility of active groups may have led to decreased adsorptive removal. Furthermore, the low efficiency of bioglass alone as an adsorbent suggests that it may have an effect on removal in synergy with biopolymer [41,42,43].

A 50 mg/L stock solution of crystal violet was prepared for the adsorption experiments. Adsorption experiments were carried out using a temperature-controlled orbital shaker (Unimax 1010, Heidolph, Schwabach, Germany). The dye concentration in the adsorption experiments was determined using a UV–Vis spectrophotometer (UV-2600, Shimadzu Corporation, Kyoto, Japan). UV-Vis analysis showed that the maximum absorption wavelength (λ_max_) of crystal violet was 590 nm. Adsorption experiments were conducted using dye concentrations of 5, 10, and 15 mg/L and adsorbent amounts of 20, 30, and 40 mg. Adsorption efficiency was calculated using Equation (1), and the adsorption value at equilibrium (q_e_) from Equation (2).

(1) $$\mathrm{Dye}\;\mathrm{removal}\;\mathrm{efficiency}\;(\%)=\frac{C_{o}-C}{C_{o}}\times 100$$

(2) $$q_{e}=\frac{\left(C_{o}-C_{e}\right)}{m}\times V$$

(C_o_: the initial dye solution concentrations (mg/L); C_e_: the equilibrium dye concentration of dye (mg/L); q_e_: the amount of dye adsorbed per unit mass of adsorbent at equilibrium (mg/g); V: the solution volume (L); m: the mass of adsorbent (g)).

## 3. Results and Discussion

### 3.1. Characterization of Sodium Alginate-Bioglass Adsorbent Spheres

**Figure 3:**
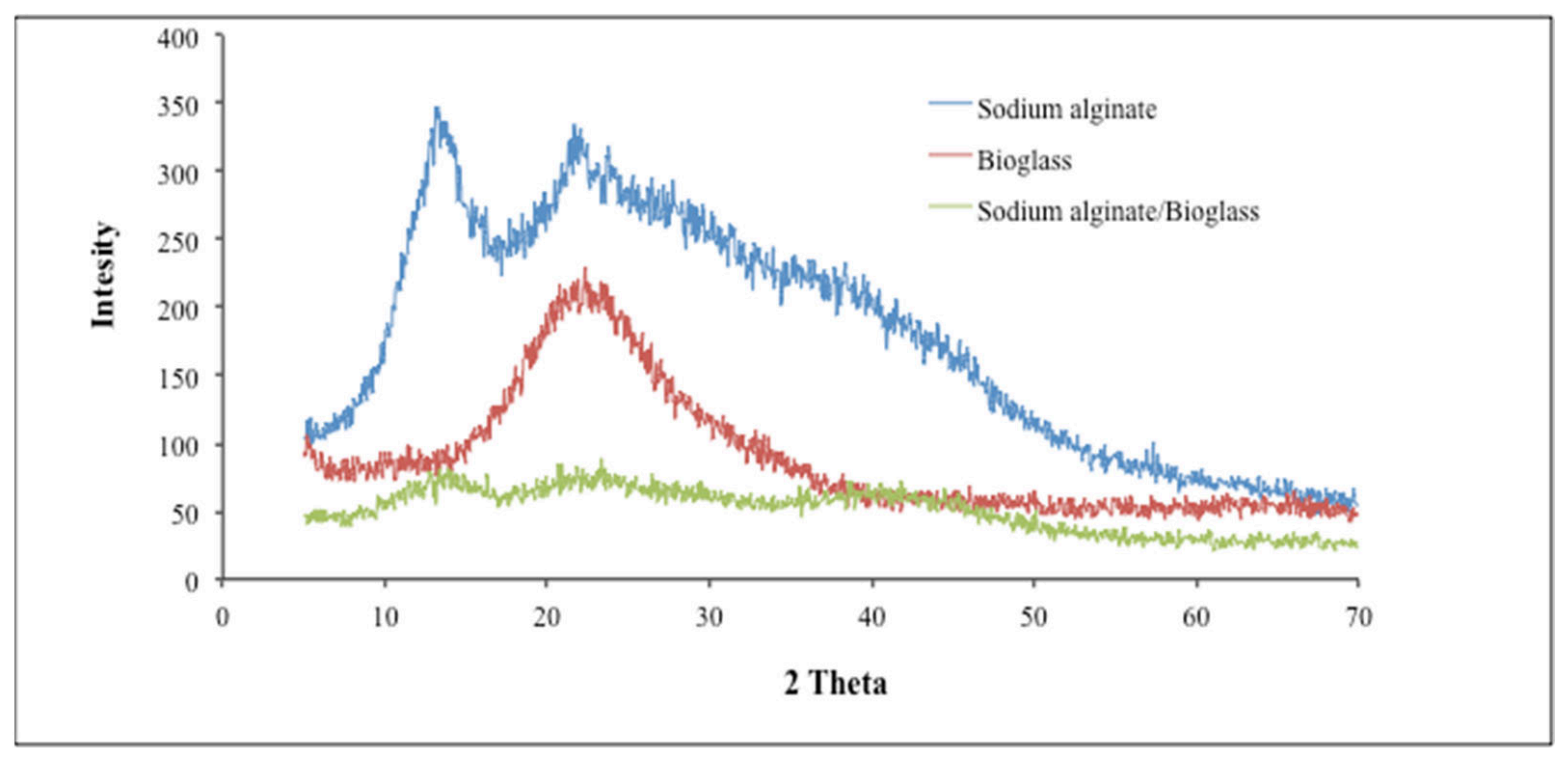
Comparison of the XRD patterns of pure bioglass, pure sodium alginate, and sodium alginate containing 14% bioglass.

XRD analysis provides information about the crystal structure of the prepared adsorbent. XRD analyses of the adsorbents prepared from SA, bioglass, and their mixture are given in Figure 3. The semi-crystalline nature of sodium alginate is indicated by peaks at 2θ = 13° and 21°, corresponding to polyguluronate and polymannuronate units. The diffraction peak at 2θ = 39° in sodium alginate is characteristic of its amorphous halo. The broad, less sharp diffraction peak at 2θ = 21° in bioglass indicates its amorphous structure. The diffraction peak intensity of the adsorbent (SA/bioglass) at 2θ = 13° has decreased. The semi-crystalline structure in SA became amorphous upon the addition of bioglass [44]. This amorphous structure may enhance surface interactions during adsorption by facilitating ion movement and interactions [45,46].

**Figure 4:**
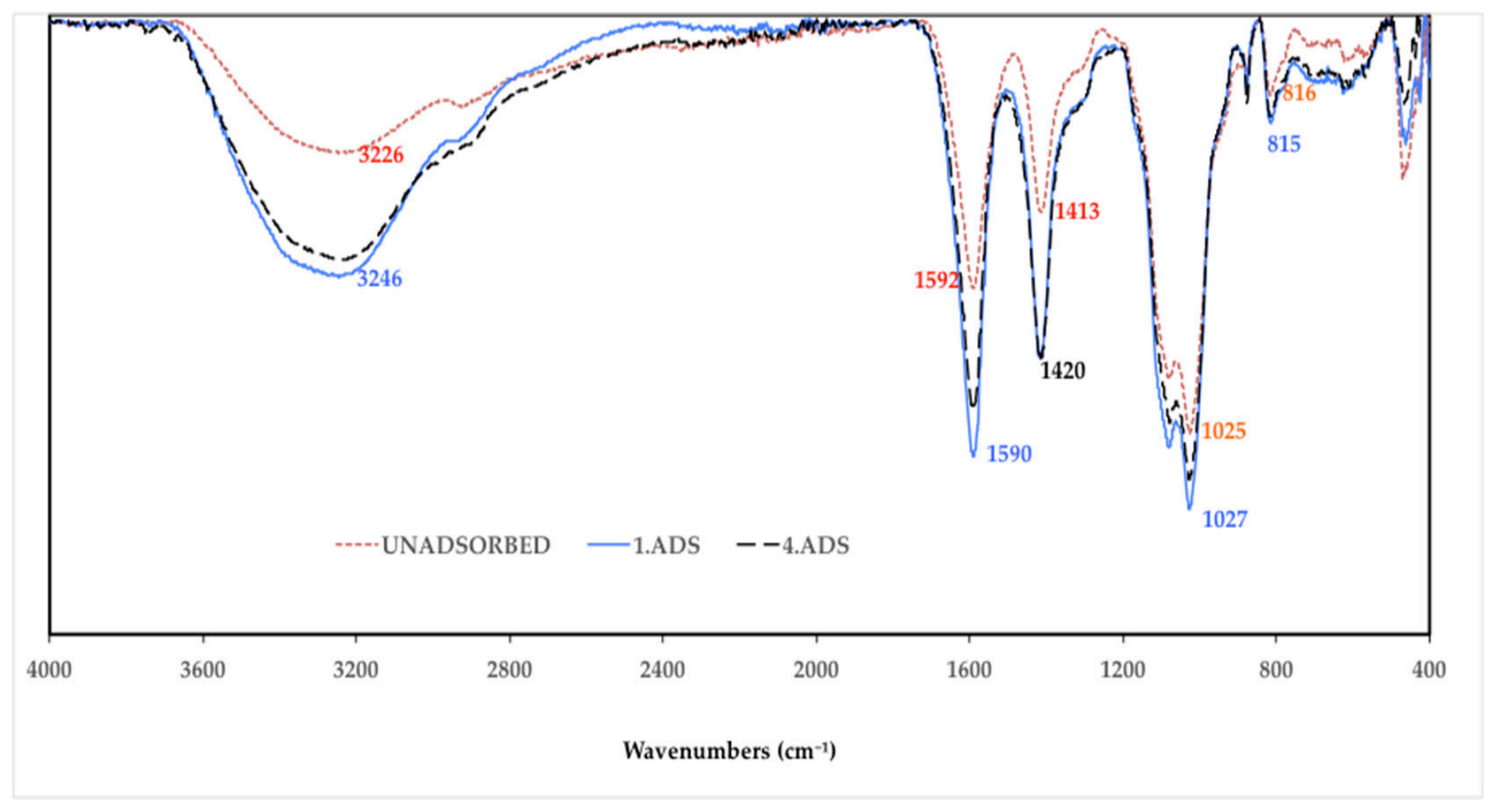
FT-IR spectra of fresh adsorbent (unadsorbed (orange)), dye loaded adsorbents (1.ADS (blue) and 4.ADS (black)).

Figure 4 shows the FTIR characterizations of the adsorbent before adsorption, after CV adsorption, and after the fourth desorption. The bands at 1592 cm^−1^ and 1413 cm^−1^ observed in the spectra are attributed to the asymmetric and symmetric stretching modes of the C–O–O bond in the alginate molecule, respectively. The peaks at wavelengths of 3226 and 1025 cm^−1^ are associated with the O-H and C-O groups in the adsorbent structure, respectively. Shifts in wavelengths are observed after adsorption. Compared with the original adsorbent (first used), the adsorbent reused after desorption exhibits a slight decrease in hydroxyl, carboxyl, and C-O functional groups. This suggests that the adsorption process may be physicochemical in nature [47]. The spectra show shifts in the dye-loaded bands. This situation can be attributed to the electrostatic binding of CV molecules to the adsorption sites. It can be interpreted as follows: the band at 3246 cm^−1^ corresponds to O-H groups participating in adsorption; the band at 1590 cm^−1^ corresponds to electrostatic interaction with (COO-) groups; the band at 1420 cm^−1^ corresponds to ionic interactions; and the band at 1027 cm^−1^ represents interactions of surface groups in the silicate structure.

### 3.2. SEM-EDX Analysis

**Figure 5:**
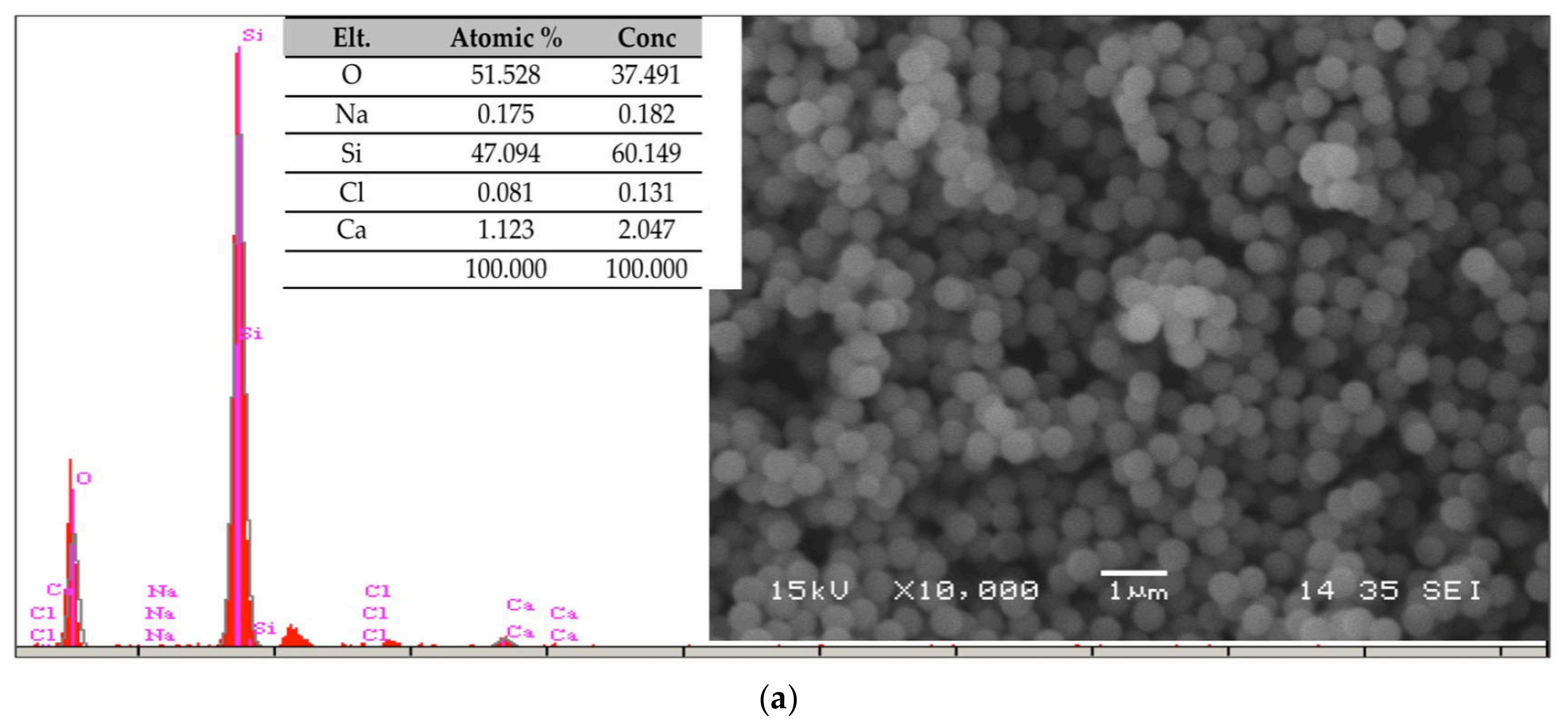

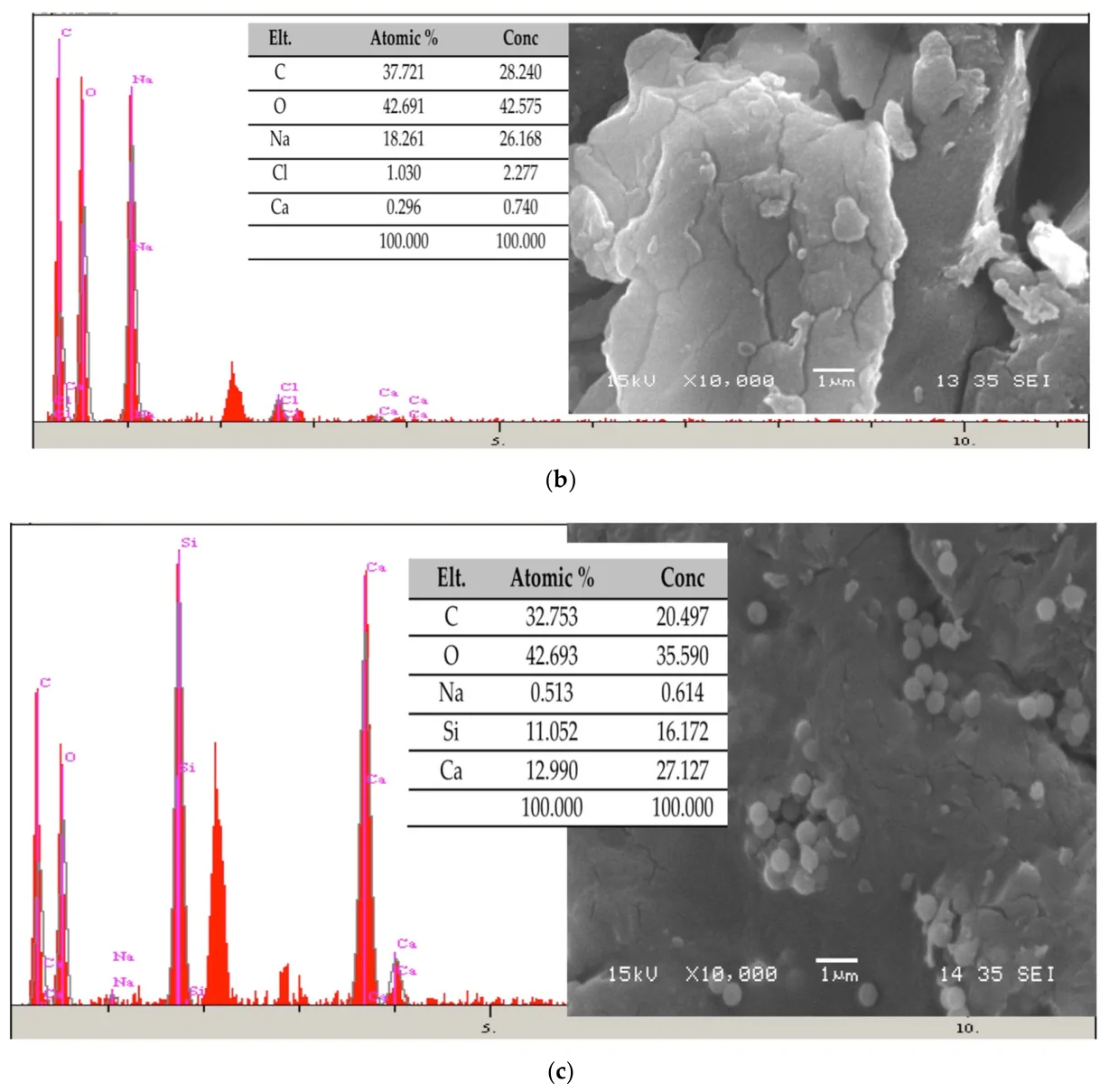
SEM images and EDX analyses of (**a**) bioglass, (**b**) sodium alginate, and (**c**) the biocomposite adsorbent.

**Figure 6:**
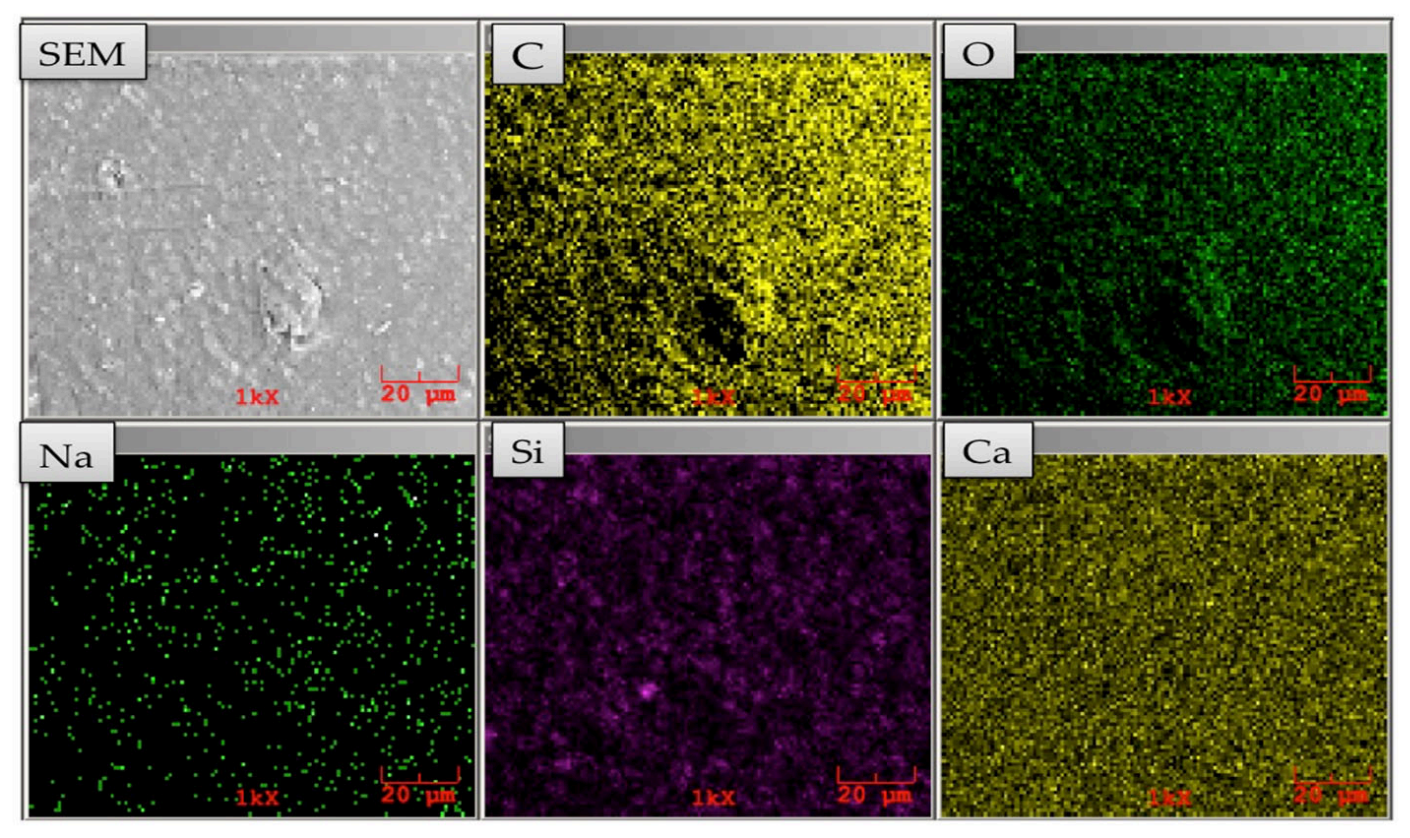
Elemental mapping of the biocomposite spheres.

SEM and EDX results of bioglass, sodium alginate, and biocomposite adsorbent are shown in Figure 5. In the SEM image shown in Figure 5a, the pure bioglass sample consists of spherical particles and exhibits high roughness. In addition, agglomerations have been observed in some areas. The close clustering of particles and high surface roughness can increase the surface area available for adsorption [48,49]. The SEM image of pure alginate shown in Figure 5b exhibited an irregular, cracked, heterogeneous morphology. This highly irregular structure can increase the surface area available for interaction between the functional groups of sodium alginate and the contaminant [50,51]. Figure 5c shows that the bioglass is well dispersed within the alginate matrix, without large-scale agglomeration. The presence of Si and Ca, as indicated by EDX analysis, demonstrates that bioglass has been successfully incorporated into the biopolymer [37,52]. EDX analysis shows that the structure contains carbon (20.49%) from the polymer, silicon (16.17%) from the bioglass, and calcium (27.12%). This demonstrates the formation of a hybrid structure, as shown in Figure 5c. Element mapping is shown in Figure 6.

### 3.3. Textural Analysis

The specific surface area and pore structure of the developed SA/Bioglass biocomposite adsorbent were examined based on nitrogen adsorption–desorption measurements. The specific surface area, total pore volume, and average pore diameter (all determined by BJH adsorption) were 7.606 m^2^/g, 0.01634 cm^3^/g, and 2.81 nm, respectively. Although the specific surface area was lower than that of conventional activated carbons, the adsorption performance of the biocomposite may depend primarily on the active sites provided by the surface functional groups of sodium alginate (including hydroxyl and carboxyl groups) and by the bioglass particles, rather than exclusively on physical adsorption arising from its porous structure.

The N_2_ adsorption–desorption isotherm curves shown in Figure S1 exhibit Type IV isotherm behavior with a hysteresis loop, supporting the presence of a mesoporous structure [53,54]. This finding is consistent with the measured average pore diameter of 2.81 nm (range: 2–50 nm). The sharp increase in adsorption at relative pressure of P/P_0_ = 0.9–1.0 indicates capillary condensation and multilayer adsorption [55]. The pore-size distribution of the developed adsorbent, shown in Figure S2, further indicates that the material is predominantly mesoporous. The sharp peak observed at 3 nm is consistent with the measured pore diameter of 2.81 nm. In addition, the second sharp peak observed at 7 nm indicates the presence of larger mesopores [53,56].

### 3.4. TEM

**Figure 7:**
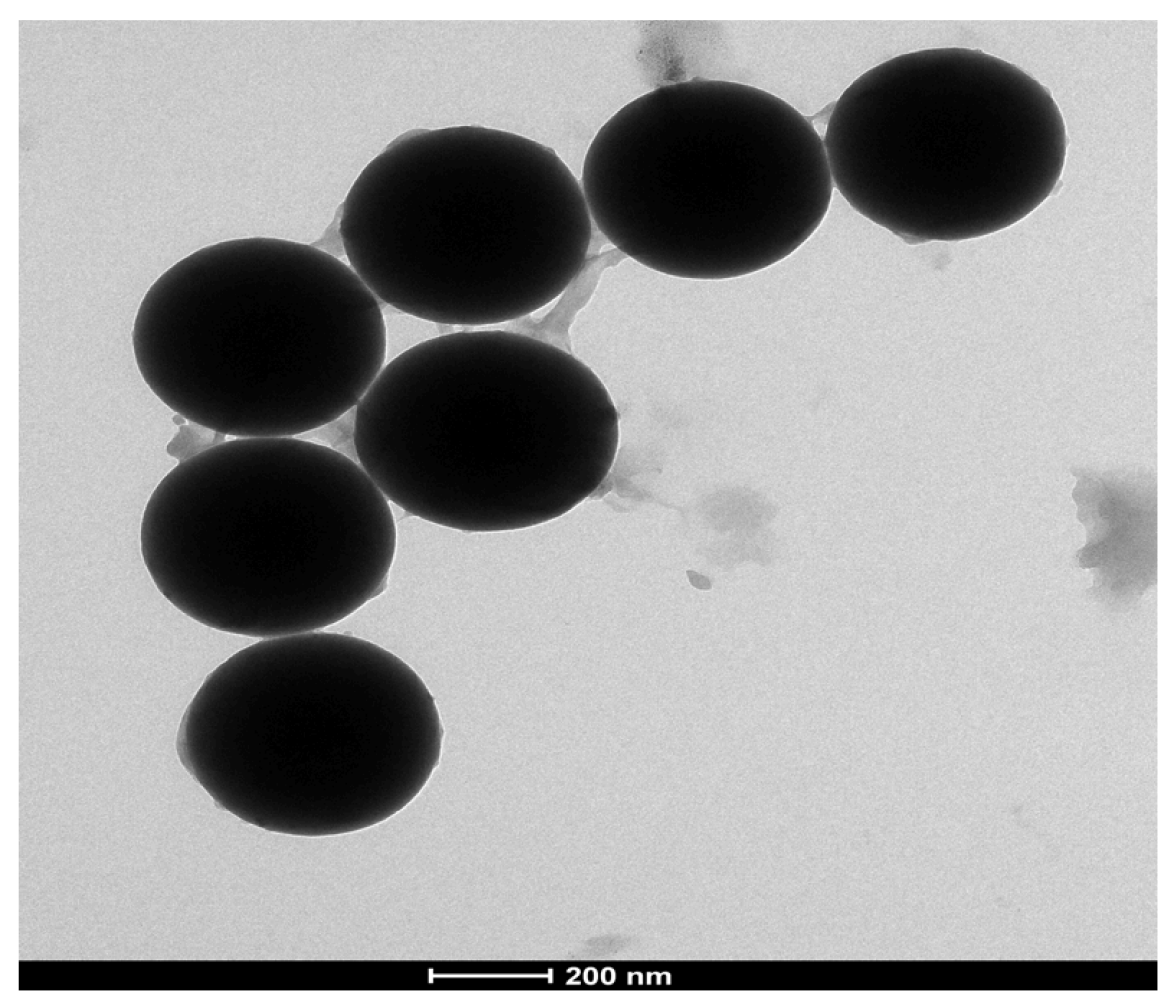
Transmission electron microscopy (TEM) image of the sodium alginate/bioglass biocomposites.

Figure 7 shows a TEM image of the ground, powdered biocomposite. The TEM image reveals nanoscale, uniform, and spherical regions within the biocomposite. In particular, spherical electron-dense regions with a homogeneous size distribution were observed [36]. These dark, electron-dense regions are attributed to bioglass-rich domains within the alginate matrix [29,57,58]. This contrast arises because bioglass contains elements such as Si and Ca, which have higher atomic numbers and electron densities than the elements predominantly present in alginate, resulting in greater electron scattering [59]. The homogeneous distribution of the dark regions indicates that the bioglass was successfully dispersed throughout the composite matrix. Such dispersion may improve the accessibility of the adsorption sites to dye molecules and facilitate the removal of crystal violet from aqueous solutions [36,60].

### 3.5. Adsorption Studies

**Figure 8:**
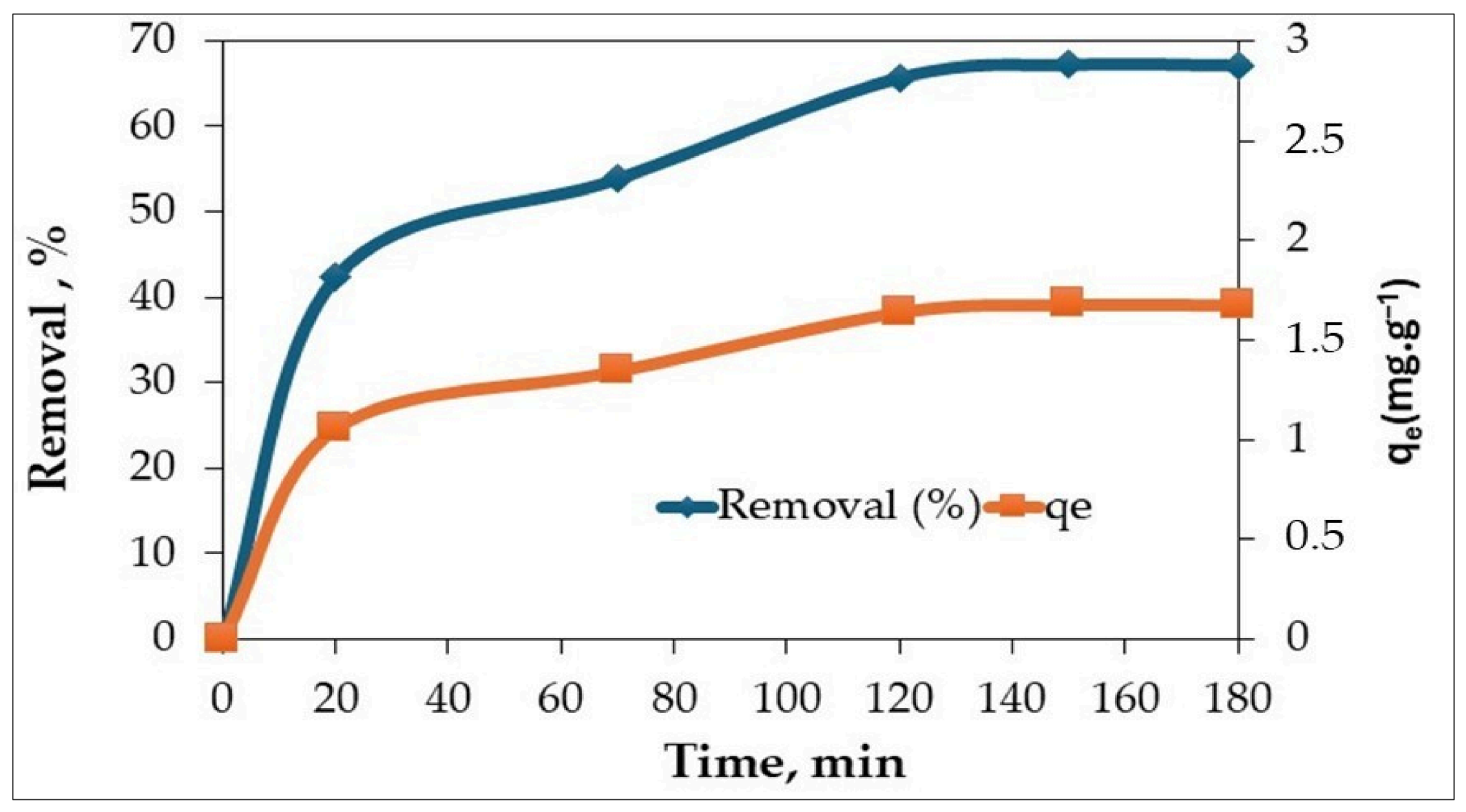
Relationship between contact time, crystal violet removal percentage and adsorption capacity.

Adsorption of crystal violet onto biocomposite adsorbent spheres was investigated by adding 40 mg of adsorbent to 20 mL of a 5 mg/L dye solution and agitating the mixture for 180 min at 298 K and 200 rpm. Figure 8 shows that the removal efficiency increased with contact time. This initial increase is due to the large number of available adsorption sites [61]. No significant increase in removal efficiency was observed after 120 min. This behavior can be attributed to the system approaching adsorption equilibrium and the progressive saturation of the active sites [62,63]. Therefore, the time required to reach adsorption equilibrium was determined to be approximately 120 min.

**Figure 9:**
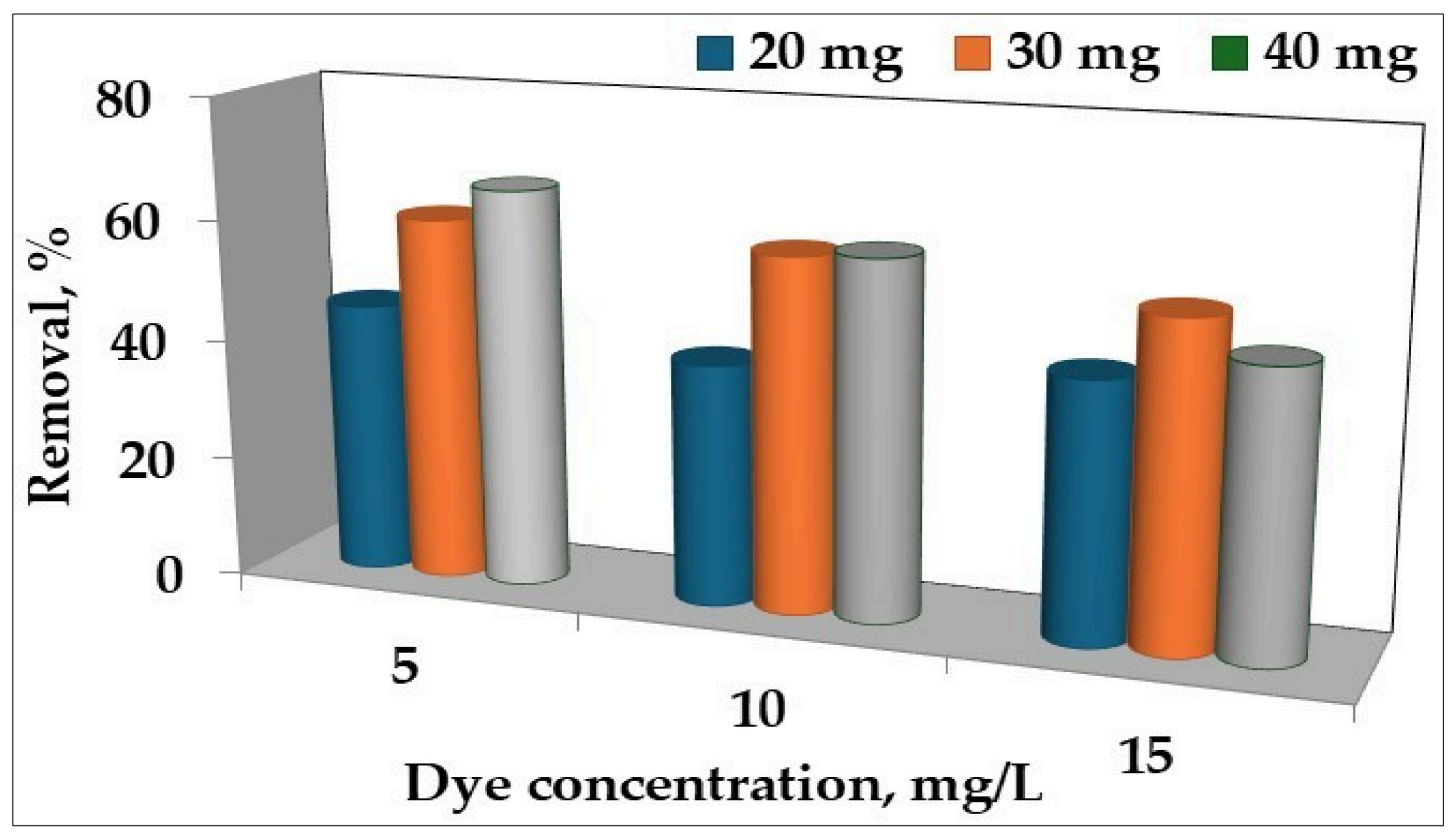
Relationship between crystal violet removal percentage and dye concentration.

Removal studies were conducted with three adsorbent amounts (20, 30, and 40 mg) in dye solutions at concentrations of 5, 10, and 15 mg/L. Figure 9 shows how removal efficiency changes with dye concentration. As shown in Figure 9, increasing the amount of adsorbent generally increased the removal efficiency. The highest removal efficiency was observed at the lowest dye concentration and the highest adsorbent concentration. This is associated with an increase in the number of active sites and the amount of functional groups available for adsorption as the dosage of adsorbent increases [64,65]. However, when the dye concentration was increased, the number of active sites required for adsorption remained insufficient, despite the increase in the amount of adsorbent [62,63,64,65,66]. Thus, removal rates decreased with increasing solution concentration. This is commonly observed in adsorption systems. Many studies report that adsorbent–adsorbate interaction is more efficient at lower concentrations and that surface saturation is delayed [67].

**Figure 10:**
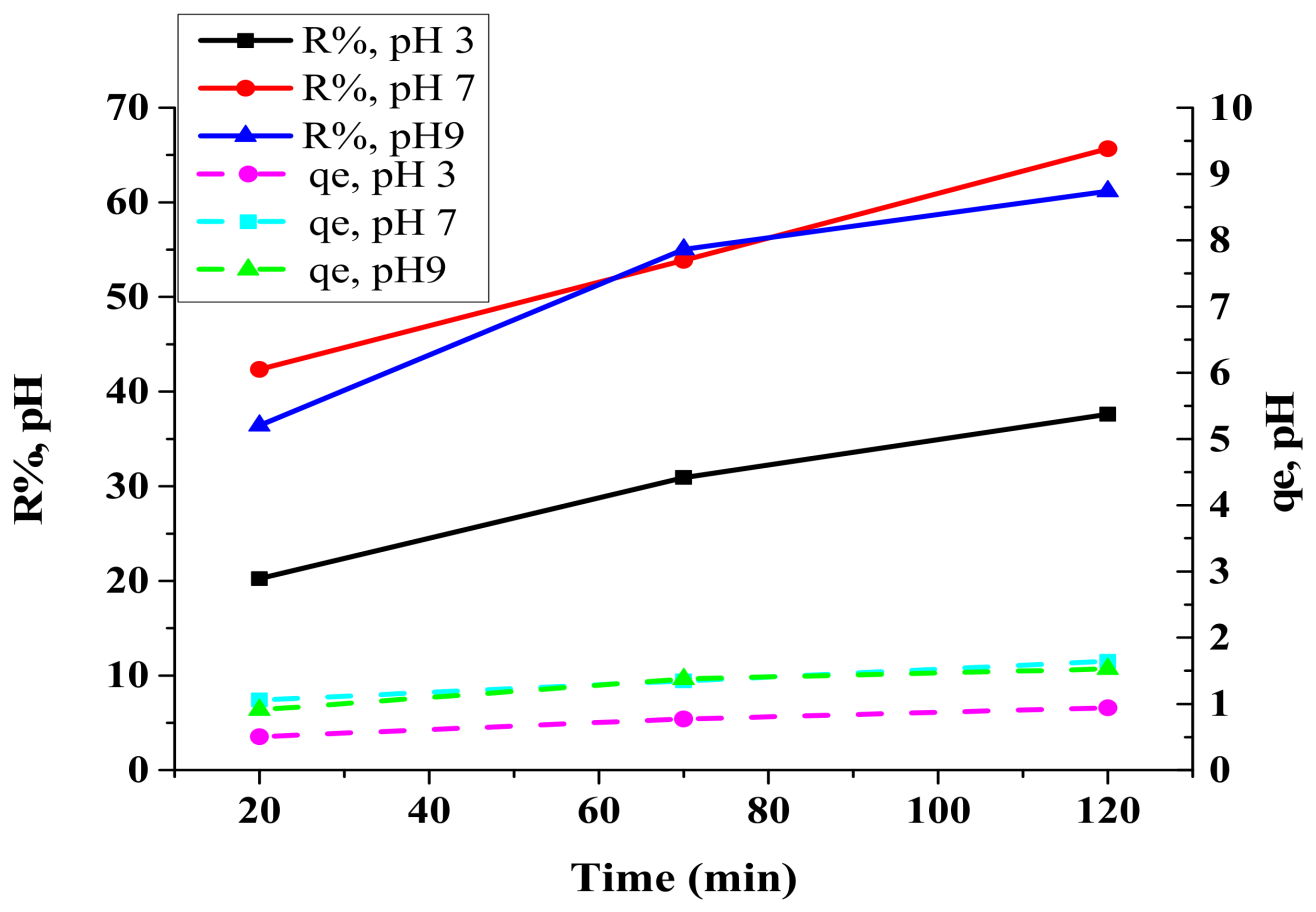
Dye removal percentage and adsorption capacity as a function of contact time at different pH values.

Figure 10 shows the effect of pH on crystal violet removal under optimum conditions. Experiments were conducted using a 5 mg/L dye solution and 40 mg of adsorbent at pH values of 3, 7, and 9. The pH of the solution was adjusted with HCl and NaOH. Removal efficiency was lower at low pH. At low pH, protonation of the adsorbent surface leads to repulsion, as the dye molecules are also positively charged. At higher pH values, the negative charge of the adsorbent increases the attraction between the adsorbent and the positively charged dye molecules [44].

In our previous study, the point of zero charge of a bioglass-loaded adsorbent used for cationic dye adsorption was found to be approximately pH 6 [44]. The natural pH of the crystal violet solution was approximately 7. Since this value is above the point of zero charge, the adsorbent surface is expected to carry a net negative charge under these conditions. Conducting adsorption at the natural pH of the dye solution may also reduce the need for chemical pH adjustment and thus improve the economic feasibility of the process. Therefore, subsequent adsorption experiments were conducted at pH 7.

For the positively charged crystal violet molecules, adsorption is favored at pH values above the point of zero charge because the adsorbent surface becomes increasingly negatively charged. The developed adsorbent is anionic, whereas crystal violet is cationic. Through electrostatic interaction, negatively charged OH^−^ ions attract positively charged dye ions, resulting in adsorption. At pH values below the point of zero charge, in the acidic pH region, excess H^+^ ions will repel the positively charged dye ions, thus preventing adsorption [68]. As shown in Figure 10, the low dye removal observed at pH 3 can be attributed to electrostatic repulsion in the acidic region below the point of zero charge. Since the highest dye removal was achieved at pH 7, which is the natural pH of the dye solution, the subsequent experiments were conducted at this pH. The higher dye removal observed at pH 9 compared with pH 3 can be attributed to electrostatic attraction under basic conditions. However, the prepared adsorbents showed a tendency to dissolve in a highly basic environment, such as pH 12 [44]. Therefore, pH values higher than 9 were not investigated in this study.

### 3.6. Adsorption Mechanism

More than one factor may influence the adsorption of crystal violet onto the developed adsorbent. Electrostatic interaction is considered to be the dominant adsorption mechanism. This interaction is caused by the electrostatic attraction between the anionic adsorbent (alginate and bioglass) and the cationic crystal violet molecules [36,69]. The hydrogen bond interaction between the OH^−^ and COO^−^ surface groups in the alginate structure and the amine groups of the dye may also contribute to the adsorption process [69,70]. In addition, bioglass may contribute to these mechanisms by increasing the surface heterogeneity and roughness of the adsorbent [36].

### 3.7. Reusability

The reusability of the adsorbent was investigated by adding 40 mg of adsorbent to 20 mL of a 5 mg/L dye solution and stirring the mixture at 298 K and 200 rpm for 120 min. Adsorption efficiency was calculated using Equation (1) in Section 2.4.

**Figure 11:**
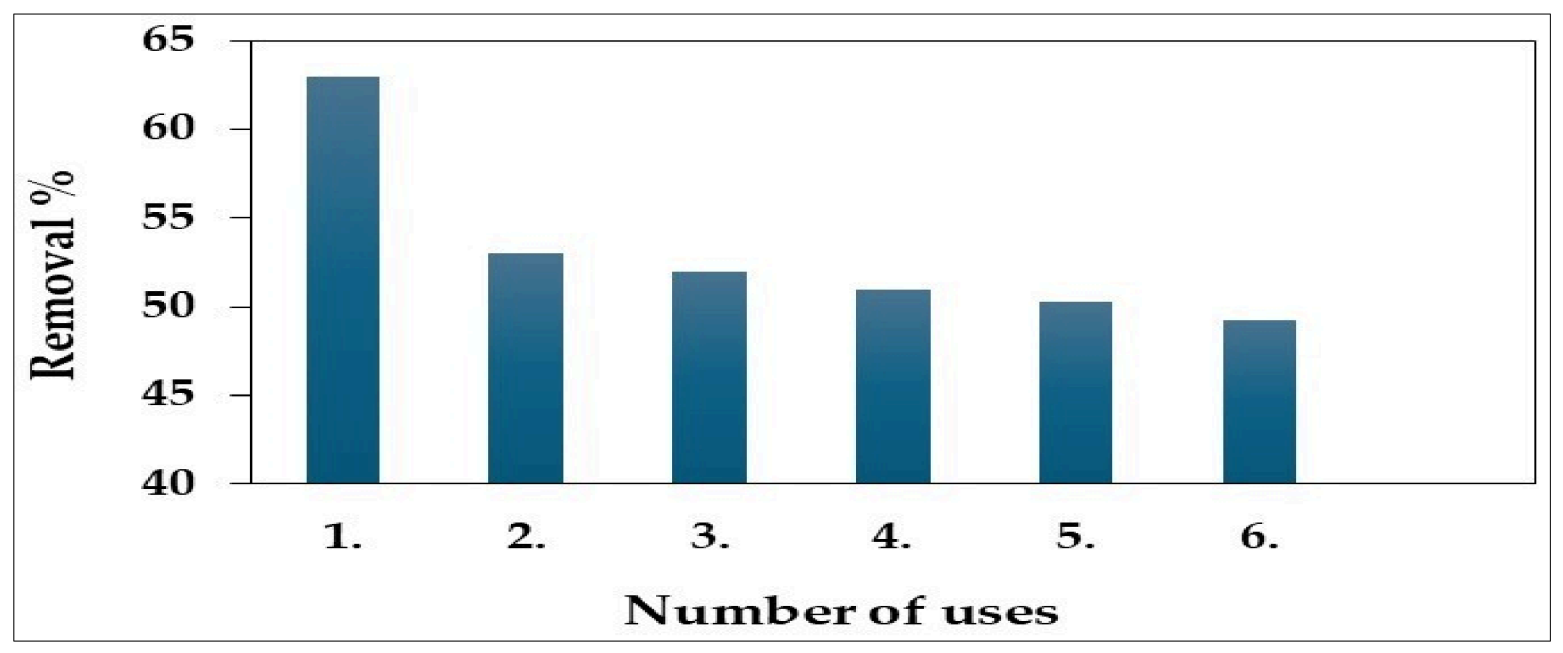
Reusability of the adsorbent.

To investigate the reusability of the adsorbent, the desorption process was carried out using an adsorbent-ethanol-water mixture after 120 min of dye adsorption. As shown in Figure 11, the dye removal efficiency remained at approximately 50% after five adsorption–desorption cycles. These results demonstrate that the prepared composite is a recyclable adsorbent with good reusability.

### 3.8. Isotherm

Isotherm studies were conducted at pH 7, with stirring at 200 rpm, using 40 mg of adsorbent and a dye concentration of 5, 10 and 15 mg/L. The adsorption capacity was determined through adsorption isotherm studies. The applicability of the Langmuir, Freundlich, Temkin, and Dubinin–Radushkevich isotherm model for crystal violet adsorption by the prepared adsorbent spheres is presented in Table 1.

**Figure 12:**
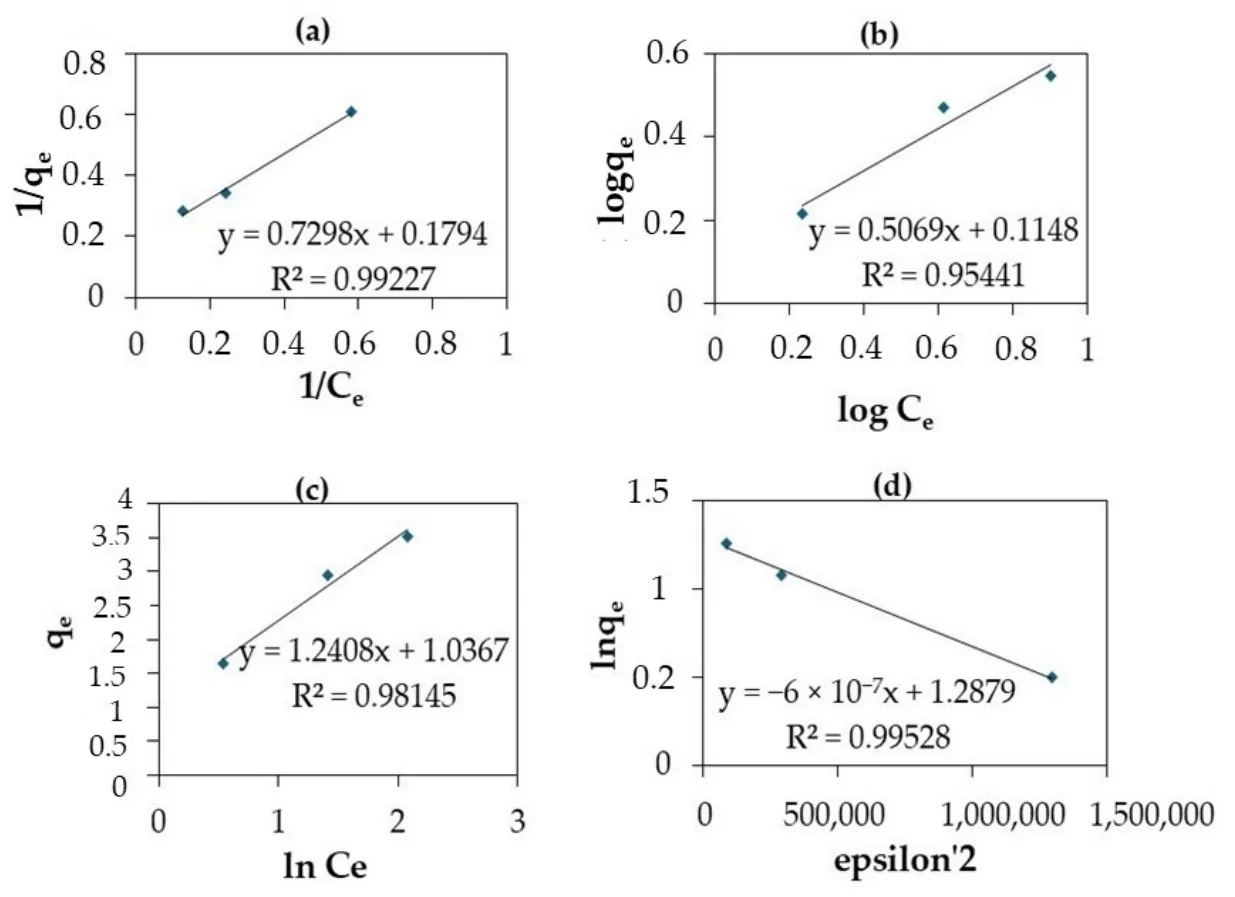
Adsorption isotherm graphs: (**a**) Langmuir, (**b**) Freundlich, (**c**) Temkin, (**d**) Dubinin–Radushkevich.

As shown in Table 1 and Figure 12, the high R^2^ values indicate a good fit between the isotherm models and the experimental data. The Langmuir isotherm assumes monolayer adsorption on a relatively homogeneous surface with a limited number of adsorption sites. The relatively high R^2^ value obtained for the Langmuir model suggests that it describes the experimental adsorption behavior well within the concentration range studied (5, 10 and 15 mg/L).

The dimensionless separation factor (R_L_) determines adsorption efficiency; availability is indicated when R_L_ is between 0 and 1. The R_L_ value was calculated as 0.45 using the equations in Table 1. The Freundlich isotherm provides information about multilayer adsorption on a heterogeneous surface. The parameters K_F_ and 1/n represent the relative adsorption capacity and the surface heterogeneity factor, respectively. The Temkin isotherm assumes that the heat of adsorption decreases linearly as the adsorbent active sites are filled with adsorbate. The average adsorption energy (E < 8 kJ/mol) calculated from the Dubinin–Radushkevich isotherm indicates that the adsorption process is predominantly governed by physisorption and that binding occurs through low-energy interactions involving weak van der Waals forces [71].

### 3.9. Kinetic

The time-dependent changes in adsorption capacity were investigated using kinetic models. The kinetic equations and corresponding parameters are presented in Table 2. The kinetic studies were performed using a solution volume of 20 mL, a solution concentration of 5 mg/L, and a mass of 40 mg of adsorbent.

The adsorption kinetics were evaluated using the pseudo-first-order, pseudo-second-order, Elovich, and Weber–Morris intraparticle diffusion models. The highest R^2^ (0.9944) was obtained for the pseudo-second-order model. Furthermore, the theoretical and experimental q_e_ values obtained from the pseudo-second-order model were in close agreement.

**Figure 13:**
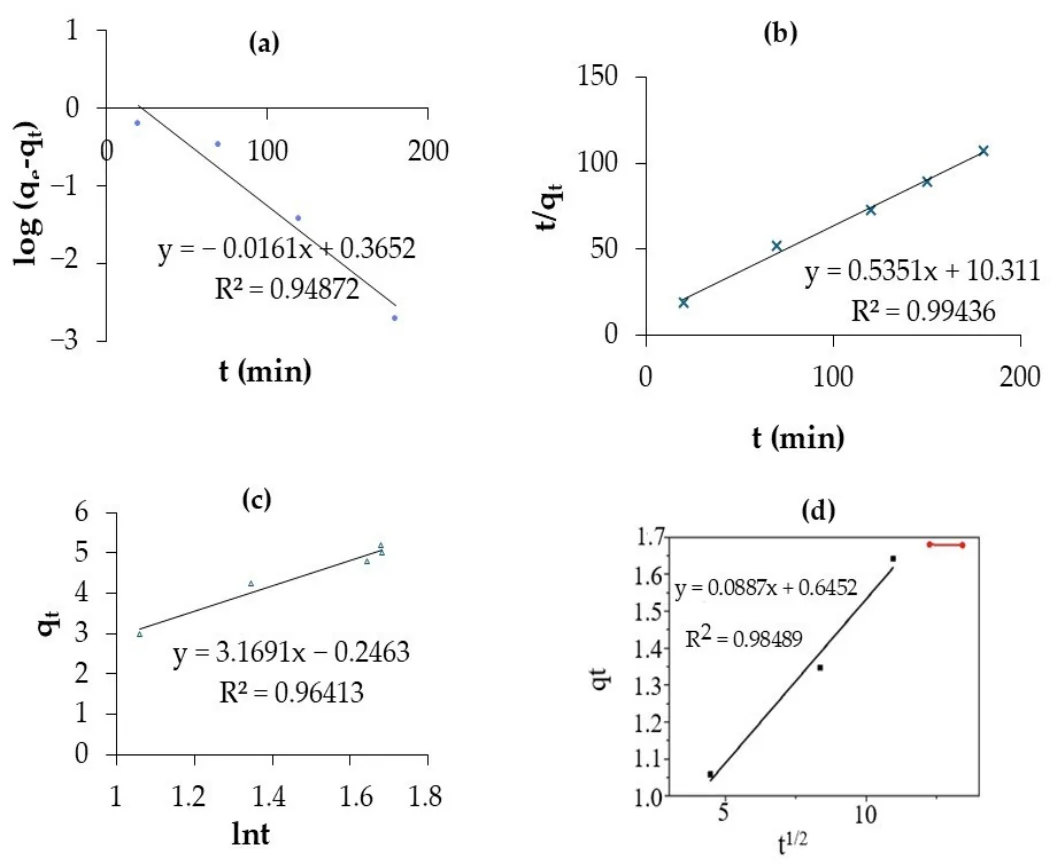
Adsorption kinetic graphs: (**a**) pseudo-first order, (**b**) pseudo-second order, (**c**) Elovich, (**d**) Weber–Morris.

The adsorption kinetics graphs are shown in Figure 13. The q_t_ versus t^1/2^ for crystal violet adsorption onto the biocomposite shows two distinct linear regions in the Weber–Morris intraparticle diffusion model (Figure 13d). The first region corresponds to external (film) diffusion, representing the transport of crystal violet molecules from the bulk solution to the outer surface of the biocomposite. The second region is associated with intraparticle diffusion, corresponding to the diffusion of dye molecules into the internal pores of the adsorbent. Since it does not pass through the origin, intraparticle diffusion cannot be considered the sole rate-controlling step. Therefore, both external mass transfer and intraparticle diffusion control the adsorption process.

### 3.10. Thermodynamic Studies

**Figure 14:**
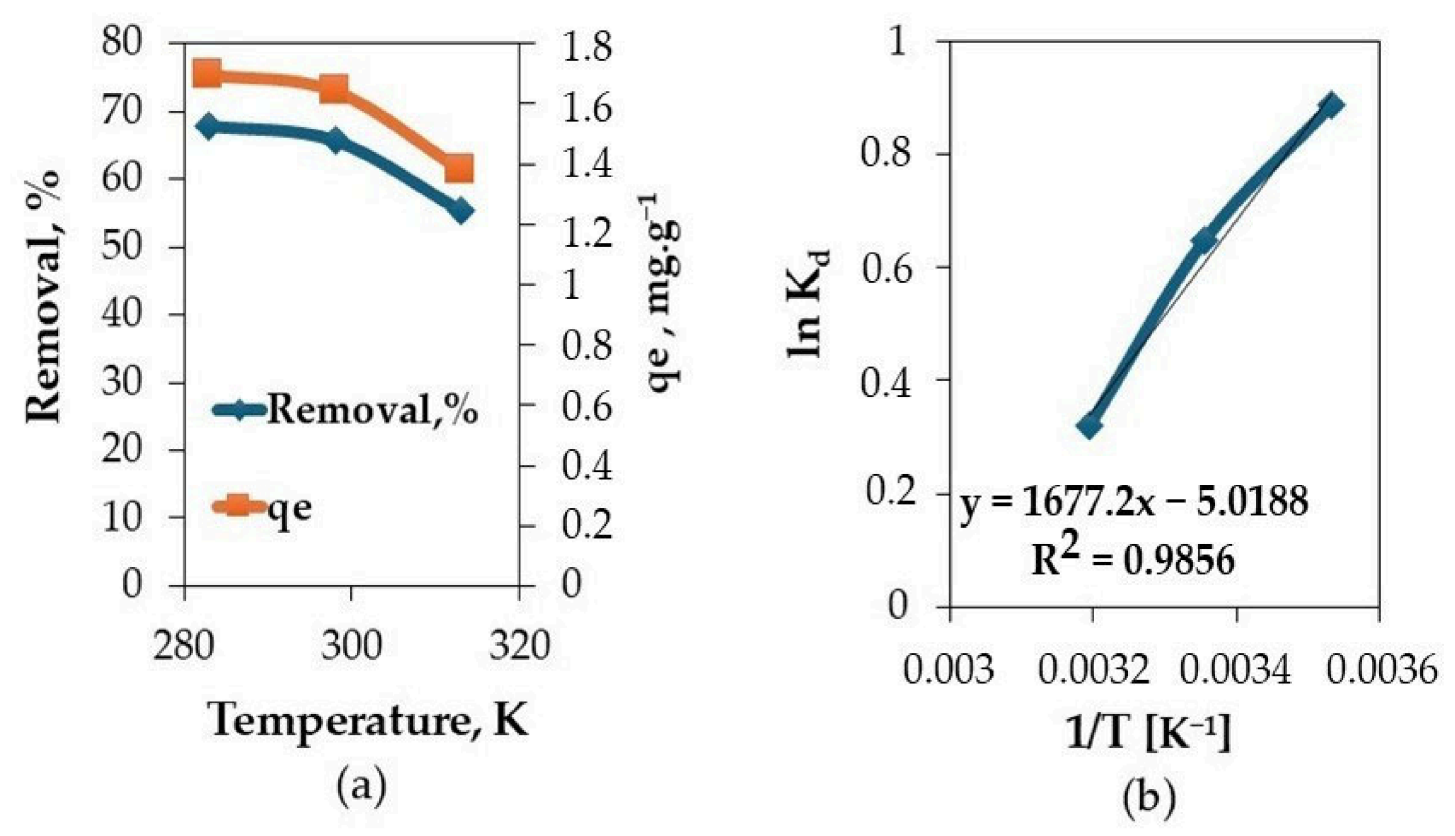
(**a**) Effect of temperature on dye removal, (**b**) Van’t Hoff equation graph.

Thermodynamic analyses were performed at 283, 298, and 313 K using a solution volume of 20 mL, an adsorbent amount of 40 mg, and a contact time of 120 min, while maintaining a constant initial dye concentration (Figure 14).

The thermodynamic feasibility of crystal violet adsorption onto the SA/bioglass adsorbent was evaluated by calculating the Gibbs free energy (ΔG°), entropy change (ΔS°), and enthalpy change (ΔH°). The equations used to calculate these parameters are shown in Table 3.

ΔS° describes the relationship at the solid–liquid interface, ΔG° indicates whether the adsorption is spontaneous, and ΔH° indicates whether the adsorption is exothermic or endothermic. A negative ΔH° value indicates an exothermic adsorption process, whereas a negative ΔS° value indicates a decrease in disorder at the solid–liquid interface.

A negative ΔG° value indicates that the adsorption process is spontaneous. As shown in Table 3, the ΔG° values became progressively more positive with increasing temperature, indicating that adsorption became less favorable and that the adsorption efficiency decreased. When the graph of ln K_d_ versus 1/T was plotted, ΔH° was calculated from the slope and ΔS° from the intercept (Figure 14b).

### 3.11. Model Fit and Optimization with RSM

RSM is a statistical methodology that improves model fitting while minimizing the number of experimental trials required to evaluate the relationship between design factors and responses. Graphical and regression analyses were performed, and a trivariate Box–Behnken design was applied using Design Expert 12 software. Three independent variables (dye concentration, adsorbent amount, and time) were tested at three levels each. The factorial points were coded as −1 and +1 (the lowest and highest parameter levels), and 0 denotes the center point. The experimental design is presented in Table S2.

Based on the removal value obtained from the experiments, the program identified the second-order (quadratic) model as suitable (Table S3) and generated the corresponding formula. Optimization values are determined using this formula. The predicted R^2^ value (0.8019) in this model is the highest value.

The model fit was evaluated using significance tests for the interactions among variables presented in Table S4. The standard deviation of the second-order model was 1.70, which was lower than those of the other models. In addition, the quadratic model exhibited the highest R^2^ (0.9876), adjusted R^2^ (0.9717), and predicted R^2^ (0.8019), together with the lowest PRESS (324.80). These results indicate that the quadratic model provides the best fit.

The significance of the model and its coefficients was evaluated using the F-values and *p*-values presented in Table S5. Both *p*-values of 0 and 0.05 are considered significant [72,73]. As the *p*-value decreases, the likelihood that a factor significantly affects the response increases. Among the significant factors, the variable with the highest F-value has the greatest influence on the response. Therefore, the *p*-value was considered first, followed by the highest F-value. A *p*-value below 0.05 indicates that the model fits the experimental data well. Table S6 shows that time had the greatest effect on the response. For the adsorption-time parameter, the *p*-value was below 0.05, and the F-value was highest.

The difference between the predicted R^2^ (0.8019) and the adjusted R^2^ (0.9717) was less than 0.2 (Table S7), indicating reasonable agreement between these values and suggesting that the model is adequate. Adequate precision was 25.9247. Since values greater than 4 are considered desirable, this result indicates that the model has an adequate signal and is suitable for design purposes.

The response under specific experimental conditions can be predicted using the developed model equations. The significance of each component is determined by comparing the corresponding coefficients. In RSM, regression equations describe the relationship between the independent variables and crystal violet adsorption. The regression equations, expressed in coded and actual values, are given in Equations (3) and (4), respectively.

(Coded) Removal (%) = 48.41 + 5.97 × A − 5.09 × B + 10.14 × C + 0.1975 × A × B + 2.85 × A × C + 3.03 × B × C − 5.90 × A^2^ + 2.49 × B^2^ − 3.82 × C^2^ (3)

(Actual) Removal (%) = −2.49160 + 3.69685 × Ads amount − 3.97570 × Dye conc. + 0.124610 × Time + 0.003950 × Ads. amount × Dye conc. + 0.005695 × Ads. amount × Time + 0.012110 × Dye conc. × Time − 0.058962 × Ads.amount.^2^ + 0.099550 × Dye conc.^2^ − 0.001526 × Time^2^ (4)

The single-factor coefficients represent the effects of the individual variables, while the multi-factor coefficients indicate the interactions among variables. In Equation (3), a positive coefficient indicates a synergistic effect, whereas a negative coefficient indicates an antagonistic effect [74]. Although both adsorbent amount (A) and time (C) positively affected dye removal, time had a greater effect than adsorbent amount.

Figure S3 presents the normal probability plot of the residuals, the residuals-versus-predicted-values plot, and the residuals-versus-run plot. Ideally, the data should lie close to the red diagonal in Figure S3a and remain within the red boundaries in Figure S3b,c.

The actual versus predicted plot is shown in Figure S4. The experimental and predicted response values lie close to a straight line, indicating good agreement between them. The 3D response surface plots and the corresponding 2D contour plots are presented in Figures S5 and S6, respectively.

### 3.12. Comparison with Literature

Table 4 presents a comparison of the crystal violet adsorption by the studied adsorbent with that of other alginate-based adsorbents reported in the literature.

## 4. Conclusions

Bioglass was incorporated into a sodium alginate solution to prepare spherical adsorbent beads for the removal of crystal violet dye from aqueous solutions. The adsorption performance of the prepared beads was investigated by evaluating the effects of adsorbent amount, dye concentration, contact time, pH, and adsorption temperature. The high R^2^ values obtained for the Langmuir and Dubinin–Radushkevich isotherm models indicate good agreement with the experimental data. The Langmuir and Dubinin- Radushkevich isotherm models both showed good agreement with the experimental data within the investigated concentration range. Among the kinetic models, the pseudo-second-order model showed the best agreement with the experimental results. Thermodynamic studies conducted at different temperatures indicated that the adsorption process was exothermic and that adsorption decreased with increasing temperature. Statistical modeling using the Box–Behnken response surface method revealed that time was the most significant variable affecting adsorption. The prepared sodium alginate-bioglass adsorbent beads can be used to remove cationic dyes. These bioadsorbents show promise for the removal of dyes from water. Although the SA/Bioglass biocomposite demonstrated effective crystal violet removal under laboratory conditions, further studies using real textile wastewater are needed to evaluate its performance in complex wastewater matrices containing competing species and various interfering components. Therefore, the current findings provide a promising basis for further development of the biocomposite as an adsorbent for treating dye-containing wastewater. Future studies could further investigate their performance in real wastewater systems and continuous-flow adsorption processes.

## Figures and Tables

**Table 1:** Relevant equations, parameters, and regression coefficients of the adsorption isotherm models.

Isotherm	Equations	Parameters	Regression Coefficients
Langmuir	q_e_ = (K_L_ q_m_ C_e_)/(1 + K_L_ C_e_) $\frac{1}{q_{e}}\;=\frac{1}{\left(K_{L}\cdot q_{m}\right)}\cdot \frac{1}{C_{e}}+\frac{1}{q_{m}}$ $R_{L}=\frac{1}{1\;+{\;K}_{L}C_{0}}$	R^2^	0.9923
		q_m_: the maximum adsorptive capacity of the bioadsorbent (mg/g)	5.57
		K_L_: the Langmuir constant (L/mg)	0.24
		R_L_: Separation factor	0.45
Freundlich	$q_{e}={\;K}_{F}{C_{e}}^{1/n}$ ${\mathrm{lnq}}_{e}={\;\ln\;K}_{F}+\frac{1}{n}{\ln\;C}_{e}$	R^2^	0.9544
		n: the constant	1.97
		K_F_: the Freundlich constant (L/g)	1.30
Temkin	$q_{e}={\;B}_{T}\;({\mathrm{lnK}}_{T}\cdot C_{e})$ $q_{e}=B_{T}{\mathrm{lnK}}_{T}+{\;B}_{T}{\mathrm{lnC}}_{e}$	R^2^	0.9815
		K_T_: the binding constant at the equilibrium (L/mg)	2.305
		B_T_: the Temkin constant (mg/g)	1.2408
Dubinin- Radushkvich	$q_{e}={\;q}_{m}\cdot \;e^{-K_{\mathrm{DR}}\cdot {ɛ}^{2}}$ ${\mathrm{lnq}}_{e}={\;\mathrm{lnq}}_{m}-K_{\mathrm{DR}\cdot }\varepsilon^{2}$ $\varepsilon =\mathrm{RTln}\;(1+1/C_{e})$ $E=1/\left(\sqrt{2}\;K_{\mathrm{DR}}\right)$	R^2^	0.9953
		q_m_ (mg/g)	3.625
		K_DR_: the Dubinin − Radushkevich constant (mol^2^/J^2^)	6 × 10^−7^
		E: the average of the adsorption energy (J/mol)	910.73

q_e_: the adsorption capacity (mg/g), C_e_: equilibrium concentration (mg/L), C_o_: initial adsorbate concentration (mg/L), R: gas constant (8.314 J.mol/K), T: temperature (K), ε: Polanyi potential).

**Table 2:** Kinetic equations and related parameters.

Kinetic Models	Equations	Parameters (C_o_: 5 mg/L, Adsorbent: 40 mg)	
pseudo-first order	${\mathrm{dq}}_{t}/\mathrm{dt}={\;k}_{1}\cdot (q_{e}-{\;q}_{t})$ $\log(q_{e}-q_{t})={\mathrm{logq}}_{e}-(k_{1}/2.303)\cdot t$	k_1_: the pseudo-first-order constant, (min^−1^)	0.0370
		q_e(theoretical)_: the adsorption capacity at equilibrium, (mg.g^−1^)	2.318
		q_e(experimental)_: the adsorption capacity at equilibrium, (mg.g^−1^)	1.642
		R^2^	0.9487
pseudo-second order	${\mathrm{dq}}_{t}/\mathrm{dt}={\;k}_{2}\cdot {\left(q_{e}-q_{t}\right)}^{2}$ $t/q_{t}=1/(k_{2}\cdot q_{e}^{2})+t/q_{e}$	k_2_: the pseudo-second-order constant, (g. mg^−1^.min^−1^)	0.0277
		q_e(theoretical)_	1.868
		q_e(experimental)_	1.642
		R^2^	0.9944
Elovich	$\frac{{\mathrm{dq}}_{t}}{\mathrm{dt}}=\alpha \cdot e^{\left(-\beta \cdot q_{t}\right)}$ $q_{t}=1/\beta \ln(\alpha \cdot \beta )+1/\beta \;\mathrm{lnt}$	α: the primary adsorption rate constant, (mg.g^−1^.min^−1^)	0.463
		β: the constant of the surface coverage size of the layer on the adsorbent, (g.mg^−1^)	3.287
		R^2^	0.9641
Weber–Morris particle intra-diffusion	$q_{t}=k_{\mathrm{id}}\cdot t^{1/2}+C$	k_id_: the Weber–Morris interparticle diffusion kinetic equation constant, (mg.g^−1^. min^−0.5^)	0.0754
		c: interfacial film resistance or boundary layer impact, (mg.g^−1^)	0.7353
		R^2^	0.984

q_t_: The time-dependent adsorptive capacity at any time (mg/g).

**Table 3:** Thermodynamic values.

Temperature, T (K)	ΔH° (kJ·mol^−1^)	ΔS° (kJ·mol^−1^·K^−1^)	ΔG° (kJ·mol^−1^)	Equations
283	−13.94	−0.0417	−2.091	ΔG°=ΔH°−T∆S°
298			−1.608	$\Delta G{}^{\circ}=-R\cdot T\cdot \ln K_{d}\;$
313			−0.829	$K_{d}=C_{a}/C_{e}$
				${\mathrm{lnK}}_{d}=∆S{}^{\circ}/R-∆H{}^{\circ}/\mathrm{RT}$

(ΔG°: Free energy difference (kJ/mol); ΔH°: Enthalpy difference (kJ/mol); ΔS°: Entropy difference (kJ/mol·K); T: Absolute temperature (K); q_e_: Equilibrium adsorption capacity (mg/g); C_a_: Concentration of adsorbate removed from the solution at equilibrium (mg/L); C_e_: Concentration at equilibrium (mg/L); K_d_: Adsorption equilibrium constant (-); R: Ideal gas constant (R: 8.314 J/(mol · K)).

**Table 4:** Comparison of the developed adsorbent with alginate based adsorbents reported in the liteature.

Adsorbent	Conditions	Adsorption Capacity or Removal Efficiency	Advantages	Disadvatages	References
Graphene oxide/SA/HEC hydrogel beads	pH = 5, 1 wt% GO, 200 mg adsorbent, 25 °C	312.72 mg/g	Green hydrogel and reusable	GO is expensive	[75]
Pullulan@sodium alginate based hydrogel	Dye conc.: 10 mg/L, 200 mg adsorbent, pH: 7, 25 °C, 210 min.	2.57 mg/g	Renewable and biopolmer-based adsorbent	Low adsorption capacity and the need for chemical cross-linking, such as with epichlorohydrin	[76]
Carbon nanotube (CNT) loaded sodium alginate beads	Dye conc.: 25 mg/L, 40 mg adsorbent, room temperature, 120 min.	32.05 mg/g 83.24%	Renewable biopolymer matrix	CNT is expensive	[31]
Pyrolyzed biochar-based sodium alginate microspheres	40 °C	223.88 mg/g	High adsorption capacity	Pyrolysis of the adsorbent at high temperatures	[77]
Alginate/PVA/Silica Hydrogel	Dual crosslinking	143.75 mg/g 69.7%	High adsorption capacity	Multi-step synthesis	[78]
Alginate/Bentonite	Acid activation	582.4 mg/g 84.59%	Very high capacity	Clay activation required	[47]
Bentonite/Alginate	Microwave	601.9	High adsorption	High temperature	[79]
Alginate/Hydroxyapatite/Calcium silicate	Ion exchange + calcination	76.8	High strength	Calcination required	[80]
Bioglass reinforced biopolymer alginate	40 mg adsorbent, 120 min, 200 rpm	5.57 mg/g 65.68%	Environmentally friendly, biocompatible, cheap	low surface area	This study

## Data Availability

The original contributions presented in this study are included in the article/Supplementary Material. Further inquiries can be directed to the corresponding author.

## Supplementary Materials

The following supporting information can be downloaded at: https://www.mdpi.com/article/10.3390/polym18172155/s1, Figure S1: N_2_ adsorption-desorption isotherms; Figure S2: BJH pore size distribution curve; Figure S3: Residual graphs: (a) normal plot of residuals, (b) relationship between residuals and predicted value, (c) relationship between residue vs. run; Figure S4: Comparison of the predicted value with the actual value; Figure S5: 3D surface graph; Figure S6: 2D contour graph; Table S1: Characteristics of Crystal Violet; Table S2: Actual and coded variables in Box-Behnken; Table S3: Fit summary; Table S4: Model summary statistics; Table S5: Sequential model sum of squares; Table S6: Determining the magnitude of the effects of adsorption parameters on removal; Table S7: Fit statistics.
