# Supplementary material for: Preparation of Biocomposite Adsorbent Spheres from Bioglass Reinforced Biopolymer Alginate and Adsorption of Crystal Violet
Source: Polymers (Basel). 2026 Sep 3;18(17):2155. doi: 10.3390/polym18172155 (PMC13568043; doi:10.3390/polym18172155)
Supplement: Supplementary file 1 [file polymers-18-02155-s001.zip › polymers-4421183-supplementary.pdf]

Supplementary material

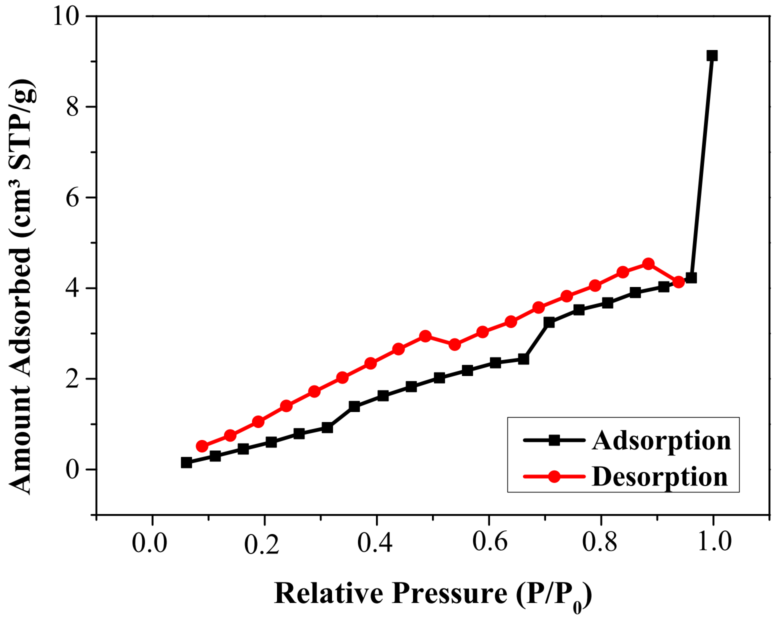

Figure S1. N_2 adsorption-desorption isotherms

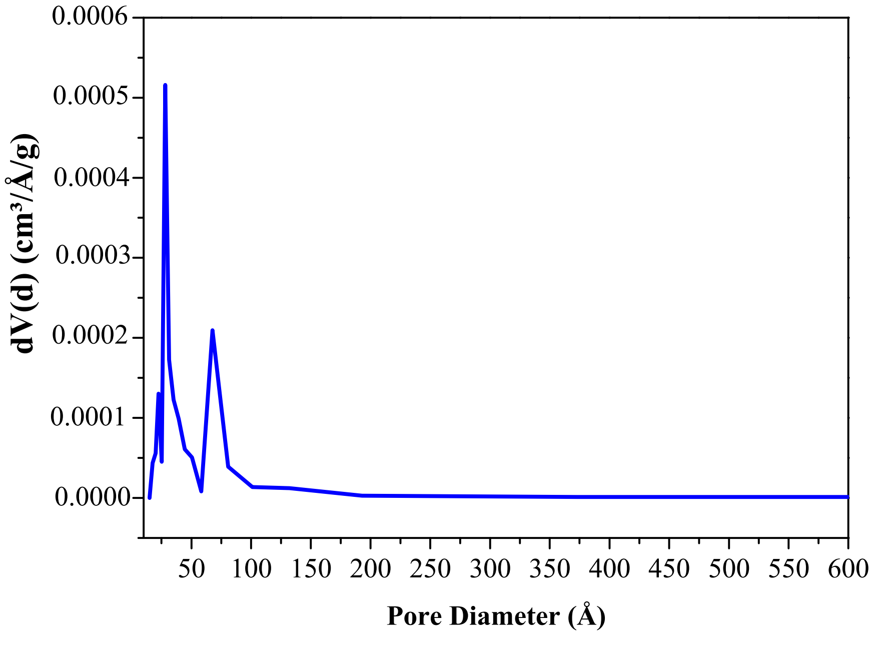

Figure S2. BJH pore size distribution curve.

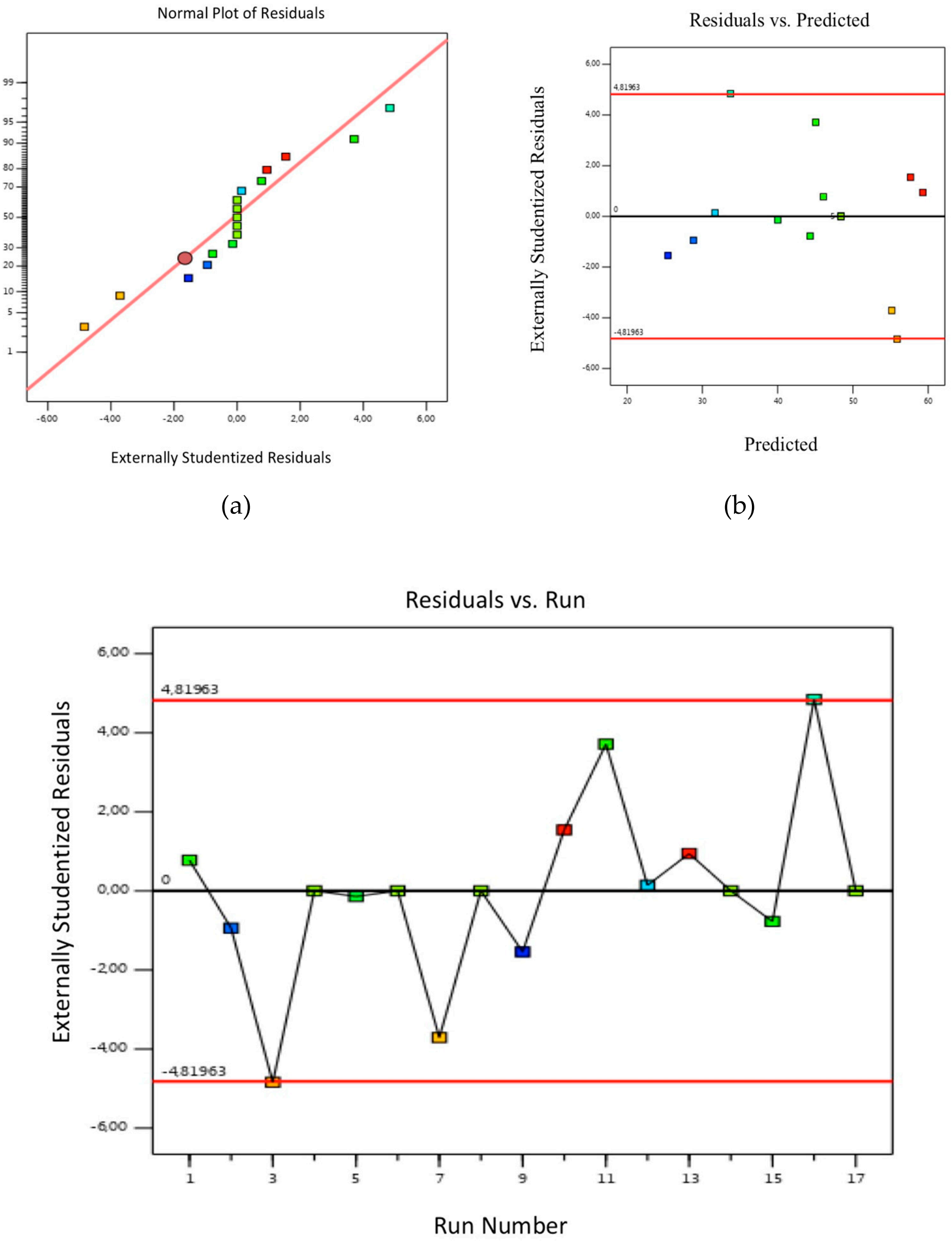

Figure S3. Residual graphs: a) normal plot of residuals, b) relationship between residuals and predicted value, c) relationship between residue vs run

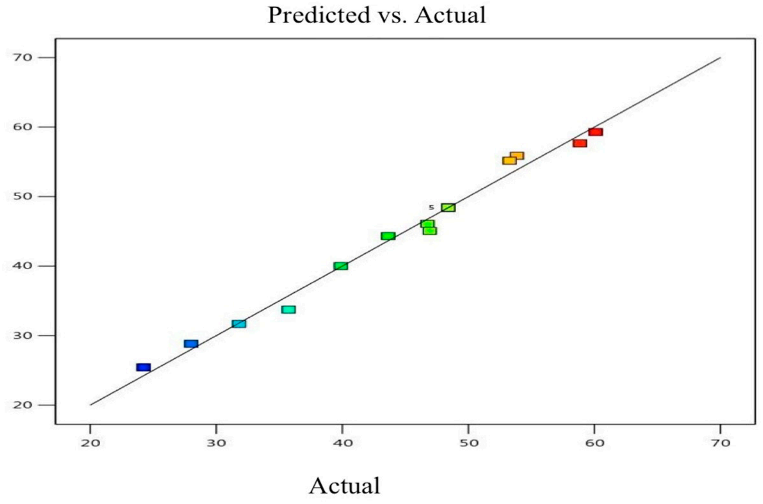

Figure S4. Comparison of the predicted value with the actual value

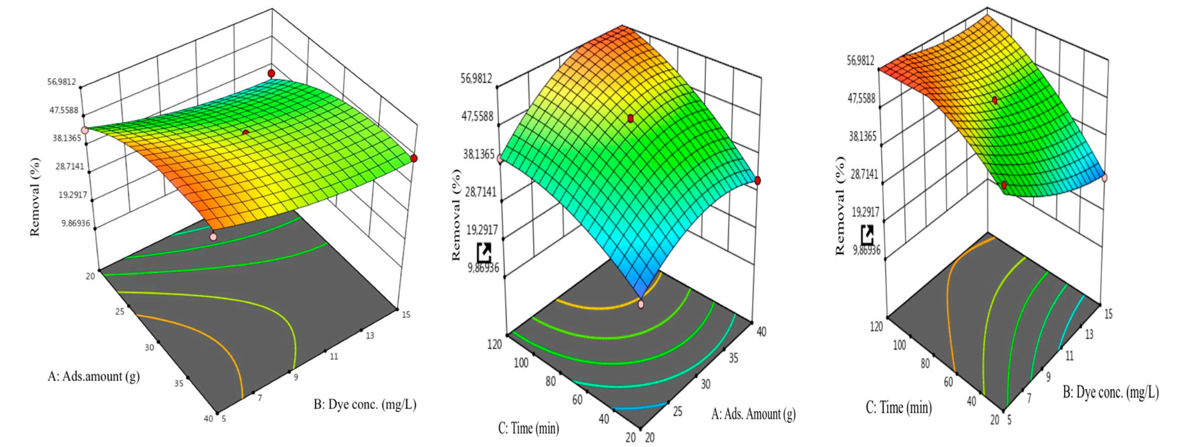

Figure S5. 3D surface graph.

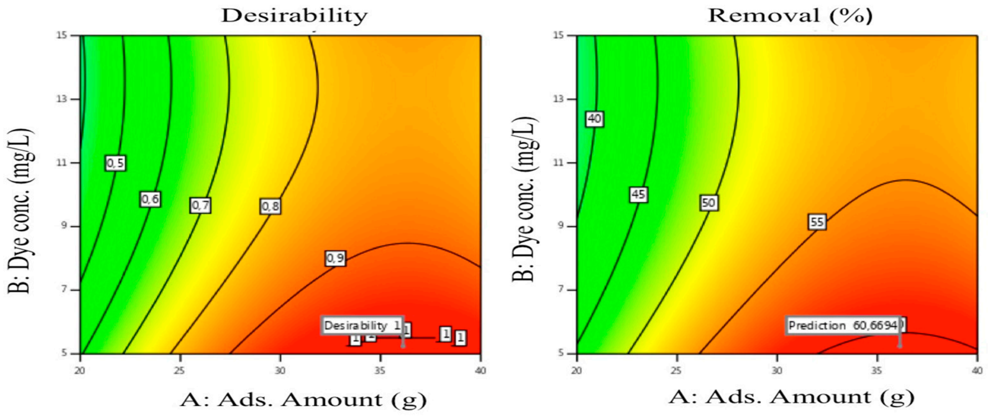

Figure S6. 2D contour graph

Table S1. Characteristics of Crystal Violet.

Chemical Formula	$C_{25}H_{30}CIN_3$
Molecular weight (g/mol)	407.99
Water solubility	4 g/L at 25°C
Ionization constant	8.64 pKa
Molecular Structure	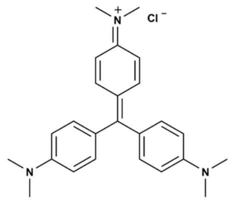
Colour Index name/ number	Basic Violet 3 / 42555

Table S2. Actual and coded variables in Box-Behnken

			Coded Levels		
Variables	Symbols	Units	–1	0	1
Adsorbent amount	A	mg	20	30	40
Concentration	B	mg/L	5	10	15
Time	C	min	20	70	120

Table S3. Fit summary

Source	Sequential p-value	Lack of Fit p-value	Adjusted R^2	Predicted R^2	Status
Linear	< 0,0001		0,7569	0,6205	
2FI	0,4720		0,7515	0,3433	
Quadratic	0,0003		0,9717	0,8019	Suggested
Cubic			1,0000		

Table S4. Model Summary Statistics

Source	Std. Dev.	R^2	Adjusted R^2	Predicted R^2	Press	
Linear	4,99	0,8025	0,7569	0,6205	622,33	
2FI	5,05	0,8447	0,7515	0,3433	1076,81	
Quadratic	1,70	0,9876	0,9717	0,8019	324,80	Suggested
Cubic	0,0000	1,0000	1,0000			

Table S5. Sequential Model Sum of Squares

Source	Sum of Sguares	df	Mean Sguare	F-value	p-value
Mean vs Total	34441,20	1	34441, 20		

Linear vs Mean	1315,80	3	438,60	17,60	< 0.0001	
2FI vs Linear	69,25	3	23,08	0,9064	0,4720	
**Quadratic vs 2FI**	234,37	3	78,12	26,94	0,0003	Suggested
Cubic vs Quadratic	20, 30	3	6, 77			
Residual	0,0000	4	0,0000			
Total	36080,92	17	2122,41			

Table S6. Determining the magnitude of the effects of adsorption parameters on removal.

Source	Sum of Squares	Df	Mean Square	F-value	p-value	
Model	1619,42	9	179,94	62,05	< 0.0001	significant
A-Adsorbent amount	285,37	1	285,37	98,40	< 0.0001	
B-Dye Concentration	207,47	1	207,47	71,54	< 0.0001	
C-Time	822,96	1	822,96	283,78	< 0.0001	
AB	0,1560	1	0,1560	0,0538	0,8232	
AC	32,43	1	32,43	11,18	0,0123	
BC	36,66	1	36,66	12,64	0,0093	
A^2	146,38	1	146,38	50,48	0,0002	
B^2	26,08	1	26,08	8,99	0,0200	
C^2	61,32	1	61,32	21,15	0,0025	
Residual	20,30	7	2,90			
Lack of Fit	20,30	3	6,77			
Pure Error	0,0000	4	0,0000			
Cor Total	1639,72	16				

Table S7. Fit statistics

Std. Dev.	1,70	R^2	0,9876
Mean	45,01	Adjusted R^2	0,9717
C.V. %	3,78	Predicted R^2	0,8019
		Adeq Precision	25,9247
